# Key tropical crops at risk from pollinator loss due to climate change and land use

**DOI:** 10.1126/sciadv.adh0756

**Published:** 2023-10-12

**Authors:** Joseph Millard, Charlotte L. Outhwaite, Silvia Ceaușu, Luísa G. Carvalheiro, Felipe Deodato da Silva e Silva, Lynn V. Dicks, Jeff Ollerton, Tim Newbold

**Affiliations:** ^1^ Department of Life Sciences, Natural History Museum, Cromwell Road, London SW7 5BD, UK.; ^2^ Centre for Biodiversity and Environment Research, Department of Genetics, Evolution and Environment, University College London, London WC1E 6BT, UK.; ^3^ Department of Ecology, Federal University of Goiás, Goiânia, GO 74690-900, Brazil.; ^4^ Centre for Ecology, Evolution and Environmental Change (CE3C), University of Lisbon, Lisbon, Portugal.; ^5^ Federal Institute of Education, Science and Technology of Mato Grosso (IFMT)—Campus Barra do Garças, Barra do Garças, MT 78600-000, Brazil.; ^6^ Department of Zoology, University of Cambridge, Downing Street, Cambridge CB2 3EJ, UK.; ^7^ Faculty of Arts, Science and Technology, University of Northampton, University Drive, Northampton, NN1 5PH UK.

## Abstract

Insect pollinator biodiversity is changing rapidly, with potential consequences for the provision of crop pollination. However, the role of land use–climate interactions in pollinator biodiversity changes, as well as consequent economic effects via changes in crop pollination, remains poorly understood. We present a global assessment of the interactive effects of climate change and land use on pollinator abundance and richness and predictions of the risk to crop pollination from the inferred changes. Using a dataset containing 2673 sites and 3080 insect pollinator species, we show that the interactive combination of agriculture and climate change is associated with large reductions in insect pollinators. As a result, it is expected that the tropics will experience the greatest risk to crop production from pollinator losses. Localized risk is highest and predicted to increase most rapidly, in regions of sub-Saharan Africa, northern South America, and Southeast Asia. Via pollinator loss alone, climate change and agricultural land use could be a risk to human well-being.

## Teaser

Pollinator losses from agriculture and climate change risk production of key crops in the tropics, especially cocoa.

## INTRODUCTION

Recent studies have highlighted rapid ongoing changes in terrestrial insect biodiversity (*1*–*3*), including among pollinating species (*4*–*8*). Some of these studies have reported net declines (*1*, *3*), while others have shown mixtures of gains and losses (*2*). Pollinator biodiversity changes have potential consequences for the provision of pollination to wild plants and crops. Evidence for insect biodiversity trends has been biased toward Western Europe and North America (*9*, *10*), with little coverage of tropical and subtropical regions (*11*, *12*). Although a few studies have shown steep declines of insects in the tropics (*3*), evidence about insect biodiversity trends there often remains anecdotal (*13*), with global syntheses (*1*, *2*) having strong geographic biases toward nontropical regions.

Among the drivers of insect and pollinator biodiversity changes, human-driven land use changes and climate change are prominent (*5*, *10*, *14*–*17*). Climate change, in particular, is emerging as an increasingly important driver (*8*, *14*, *18*–*21*), although among taxa responses are likely mixed to some extent (*22*). Synergistic interactive effects of land use and climate change are often associated with further reductions in insect biodiversity compared to if the pressures acted in isolation (*23*–*27*). A key mechanism underpinning interactive land use and climate effects is the altered microclimatic conditions in areas where vegetation has been modified for human land use (*23*). Tropical insects are expected to be more susceptible to climate change, including interactive effects with land use, given their narrower physiological tolerance compared to nontropical species (*28*). Recent studies show greater effects in tropical than nontropical insect biodiversity (*27*).

Changes in the biodiversity and composition of pollinator communities are expected to have large effects on the provision of pollination services. Pollen limitation from animal pollinator losses has already been shown to reduce the reproductive success of wild plants (*4*, *29*) and the productivity of certain crops (*30*–*34*), although there is no clear evidence that pollen limitation is yet causing wholesale reductions in yields of crops that rely on animal pollination (*35*). Evidence that insect biodiversity responds to human pressures more strongly in the tropics than elsewhere (*17*, *27*) is noteworthy, given that the majority of animal pollination–dependent crops are grown in the tropics (*36*). However, it is not only tropical countries that will experience the effects of pollinator losses and subsequent pollen limitation, with high-income countries benefiting from imports of animal pollination–dependent foods from tropical areas (*37*). Abundance, species diversity, and functional diversity of pollinators have all been implicated as determinants of the delivery of pollination service (*33*, *38*–*42*). Previous attempts to model the provision of crop pollination service are often based on predictions of pollinator abundance, which bears a direct relation to pollination of plants, and has been shown to give a reasonable approximation of pollen deposition in at least some study systems (*43*). A key uncertainty however relates to the shape of the functional relationship between pollinator abundance and crop production (*43*).

Here, we present a global assessment of the interactive effects of climate change and land use on pollinator abundance and predictions of how the inferred abundance changes might translate into risk to crop pollination worldwide based on a range of possible abundance-pollination relationships. Because species richness has also shown to be important for provision of crop pollination, we also tested the robustness of our models and projections to using Chao-estimated species richness in place of total abundance (*44*). Our underlying analyses are based on a space-for-time framework using the PREDICTS (Projecting Responses of Ecological Diversity In Changing Terrestrial Systems) database of biodiversity recorded in different land uses (*45*), together with a list of species identified in the literature as likely pollinators (*17*). We use mixed-effects models to fit total pollinator community abundance as a function of land use (primary vegetation versus cropland) and a measure of historical temperature change between a baseline period (1901–1930) and the year before the end month of biodiversity sampling (see fig. S21 for the duration of each sampling period), standardized by monthly temperature variability in the baseline period (*27*, *46*). We standardized temperature changes in this way to capture where temperatures have exceeded the baseline seasonal and interannual variation, a consideration that has previously been identified as important for insects in general (*27*). Given that the PREDICTS database contains a set of biodiversity measurements collected from across studies, we include a set of random intercepts to account for study-level variation, as well as an adjustment for sampling effort. Our set of likely insect pollinators was compiled through an automatic literature scrape, combined with a manual search and verification by a group of pollination ecology experts (*17*). We then apply these models to predict which locations and crops are likely to be exposed to the greatest losses of pollinator abundance and thus to face the greatest risk of crop pollination shortfalls. We moderate estimates of risk according to estimates of where crops are grown (*47*), how dependent these crops are on animal pollination (*36*), projections of historical and future climate change, and a set of assumptions for the relationship between local pollinator abundance and crop pollination (from linear to highly concave to convex). We focus on future climate projections under two Representative Concentration Pathway (RCP) scenarios: RCP 2.6, which has a multimodel median–predicted 1.5°C increase in global average temperatures by 2100 compared to the preindustrial climate, and RCP 6.0, which has a multimodel median–predicted 3°C increase in global average temperatures (*48*). Last, we combine projected pollination risk with estimates of the trade in pollination-dependent crop production (*37*) to predict regions of the world that may be vulnerable to the indirect consequences of crop pollination risk via trade connections.

## RESULTS AND DISCUSSION

**Fig. 1.**
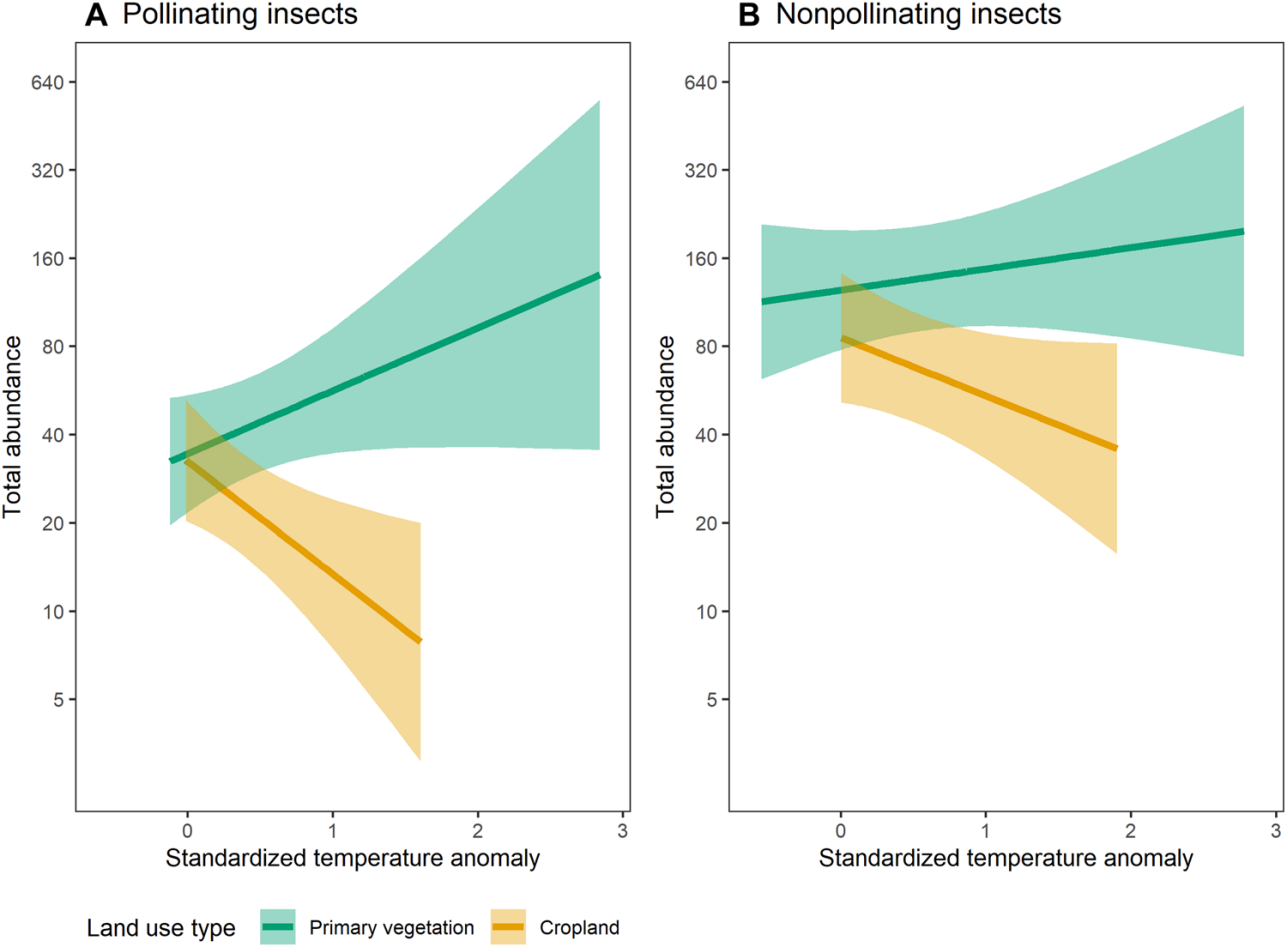
Response of pollinating and nonpollinating insect total abundance to the interactive effect of standardized temperature anomaly and land use. (**A**) Pollinating insects: *F* = 22.4068, *P* < 0.001; (**B**) Nonpollinating insects: *F* = 10.7520, *P* < 0.01. Note that abundance is plotted on a log_e_ scale (although the labels are back-transformed). Results are based on linear mixed-effects models. Site numbers are as follows (also see table S1 and fig. S14 for site spatial distribution): insects known to pollinate (primary vegetation = 1166, cropland = 1507); insects not known to pollinate (primary vegetation = 1747, cropland = 922). See table S2 for the number of species represented in both the pollinating and nonpollinating groups, table S3 for AIC and *R*^2^ values for each model, figs. S18 and S19 for the number of insect pollinating species, fig. S20 for the same figure but with the values of total abundance, and fig. S1 for models by taxonomic order. Shading represents 95% confidence intervals around the mean fitted effect. Green, primary vegetation; yellow, cropland.

The abundance of insect pollinators responded strongly to the interaction of recent climate change and land use (Fig. 1). Within cropland experiencing novel temperatures (standardized temperature anomaly = 1), pollinator abundance is 61.1% lower than in natural habitat that has not experienced temperature increases. The causal mechanism underpinning this interaction is unclear, but the moderation of microclimatic conditions (*49*) and the absence of a buffering effect of forest within cropland (*50*), are likely partly responsible. Our results are qualitatively consistent with recent results for a sample of all insects (*27*), but importantly we show that responses to the interactive effects of climate change and land use are stronger for pollinating than nonpollinating insects (Fig. 1). This is important, as it would indicate that risk inferred from all insect groups will be lower than from insect pollinating taxa alone. We also show that the strength of the interactive effect varies among taxonomic groups, with the strongest effects seen in dipteran and hymenopteran pollinators (fig. S1). Whether the sensitivity of pollinating insects to the interaction of climate change and land use relates directly to their reliance on floral resources or to other correlated traits typical of pollinators is unclear, and a combination of both factors is likely to operate. For example, selection of animal pollinated plants is thought to be highly sensitive to climatic conditions such as precipitation and temperature (*51*), suggesting that plant-pollinator interactions are highly sensitive to thermal changes. Pollinator pilosity (i.e., hairiness), on the other hand, likely affects an insect’s ability to adapt to changes in climate (*52*) and, given its nature as a trait typical of bees and hoverflies, tends to be correlated with a reliance on floral resources (*53*).

The modeled effects of land use–climate interactions on pollinator abundance are robust to using threshold temperatures to restrict the months considered to those in which insects are likely to be active (fig. S3), to jack-knifing the predictions for the top 10 sampling methods (fig. S22; see fig. S23 for the full set of sampling methods), and to including an interaction between mean annual temperature and predominant land use (table S6; also see fig. S24, showing the correlation between standardized temperature anomaly and mean annual temperature, and fig. S25, suggesting that mean annual temperature may have an effect on primary vegetation but not in cropland). Although the lepidopterans are the only group represented for a standardized temperature anomaly greater than 2 (fig. S1), our results are robust to dropping individual taxonomic families from the model dataset (fig. S2).

**Fig. 2.**
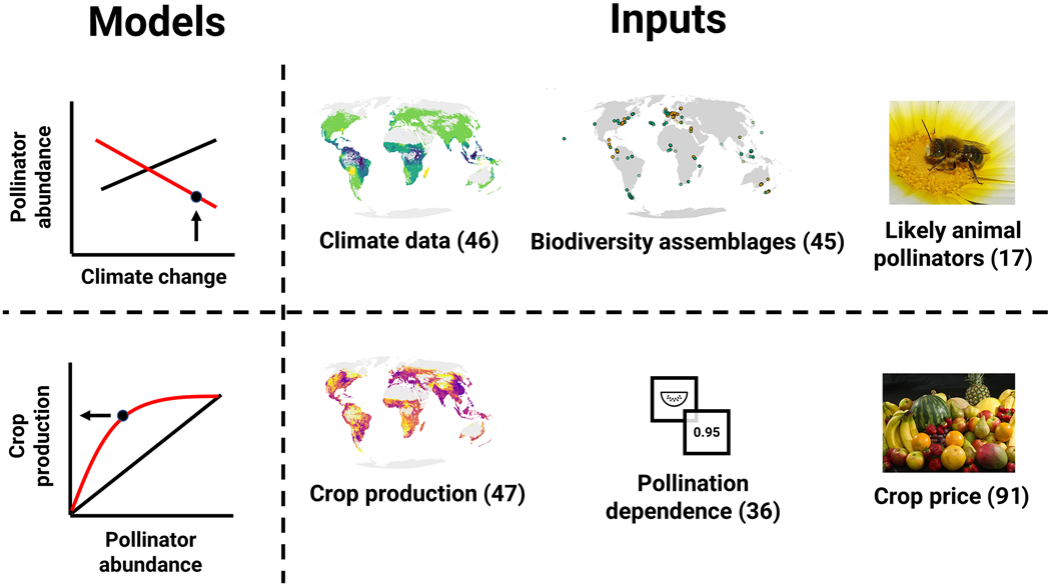
A schematic of models and inputs for our local production risk measure. (**Top**) The PREDICTS database (“Biodiversity assemblages,” subset for a set of “Likely animal pollinators”) is used to build a space-for-time model of pollinator biodiversity change, fitting total abundance as a function of an interaction between land use type and a standardized temperature anomaly of climate change (“Climate data” from the Climatic Research Unit). (**Bottom**) Change in insect pollinator abundance relative to a baseline (where standardized temperature anomaly is 0) is converted into a crop production loss via a set of linear functions and then converted into a pollination-dependent risk by multiplying the expected change by the pollination-dependent production in each cell (“Crop production” and “Pollination dependence”). Economic loss is calculated from production at risk multiplied by crop price. Numbers in brackets for each input represent the source from which the data originated (as listed in the bibliography). Image credits: Bottom right photo, Ionutzmovie (CC BY 3.0); top right photo, gailhampshire (CC BY 2.0).

**Fig. 3. F3:**
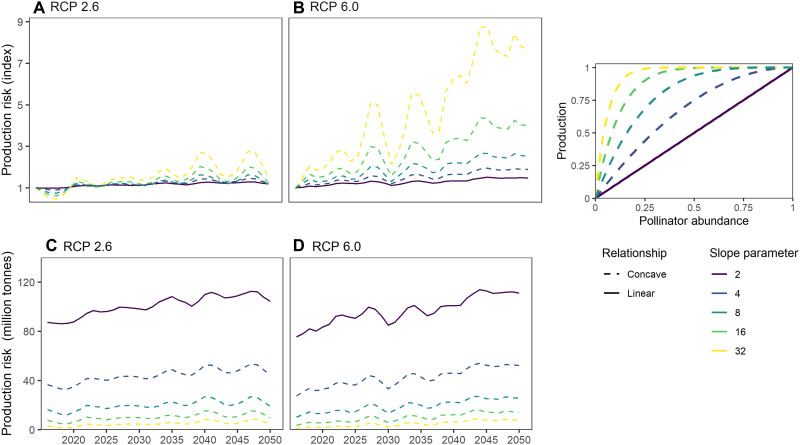
Projected change in total production risk under two RCP scenarios (2.6 and 6.0; see fig. S7 for 8.5) and a set of hypothetical relationships between pollinator abundance and crop production (linear and varying degrees of concavity, defined in the righthand panel). Results are shown both for an index of change in relative risk (**A** and **B**) and for the total production potentially at risk (in tonnes) (**C** and **D**). For each year into the future, the standardized temperature anomaly was projected globally for all cells with production of crops dependent on animal pollination using a 3-year rolling average of temperature anomaly estimates in each cell, from an ensemble of different climate models. We used data on crop production for the year 2000 (the latest year when such data are available for all crops). For each annual projection of standardized temperature anomaly, insect pollinator abundance on cropland was predicted according to the model shown in Fig. 1A and then expressed as proportional abundance loss compared to cropland that has experienced no warming (i.e., standardized temperature anomaly of 0). In each cell, the total production of each crop (*47*) was multiplied by dependence on animal pollination (*36*) and then adjusted for the predicted percentage reduction in insect pollinator abundance in that cell. These estimates of crop production at risk were summed across crops and then across all terrestrial cells globally. Lines show different assumed relationships between insect pollinator abundance and crop production: dashed, concave relationships (of differing degrees, indicated by different colors: yellow, most concave; purple/blue, least concave) and solid, linear relationship. See fig. S7 for a set of convex relationships.

We predict that total pollination production risk will increase under all climate scenarios ([Fig F3]see Fig. 2 for a schematic of our crop pollination risk inputs and models, and Fig. 3 for the change in risk). For all scenarios, our projections use change in pollinating insect abundance as a proxy for the relative risk to the production of crops dependent on insects, incorporating information on where crops are grown worldwide (*47*), as well as the fractional dependence of crops on animal pollination (*36*). Our projections are based on the assumption that a projected loss in pollinator abundance will be associated with risk to crop production from loss of pollination services, according to a function that translates abundance loss into production loss. This linked cascade model allows us to account for uncertainty of the biodiversity-production relationship, which, although typically described as positive concave, may vary for crop-pollinator interactions (*54*). We made a decision to focus on abundance in the main text given the mechanistic link between pollinator abundance, pollen load, pollen deposition, and crop pollination. To be complete, however, in the Supplementary Materials we also present a measure of risk based on species richness alone (figs. S15 and S16). Our estimates of both relative risk and absolute production risk should be interpreted as indices of risk, rather than predictions of the actual amount of production likely to be lost, given the very high uncertainty in how pollinator abundance changes will translate into actual production losses (*55*) and that we do not account for the spatial context of individual cropland areas.

Increases in risk are seen for all assumed relationships between abundance loss and production risk, although the magnitude of changes in relative risk and especially absolute production risk varied widely (Fig. 3). The predicted rate of increase in average production risk was substantially higher under RCP 6.0 than RCP 2.6, suggesting that efforts to mitigate climate change will reduce the risk to future crop production, alongside the many other benefits (*56*). Relative production risk varied strongly between years under an assumption of a concave relationship between pollinator abundance and production (Fig. 3). This volatility may be explained by the way in which the nonlinearity of abundance/production relationships interacts with interannual climate variability caused by the El Nino Southern Oscillation (*57*). While increasingly concave assumed relationships between insect abundance loss and crop production risk led to steeper increases in relative production risk with future climate change, absolute production risk was markedly lower, owing to a lower baseline in the present day (Fig. 3). Our estimates of risk are based on the distribution of crops as grown in 2000 (see table S4 for the full list of crops), meaning we do not account for changes in the distribution of crops over time, which are likely to occur as a result of the direct impacts of climate change, indirect effects through the loss of pollinator biodiversity, and socioeconomic factors such as price changes in the global markets for particular crops.

Our projections of crop production risk are robust to variation in climate predictions under different individual climate models (fig. S4) and do not change markedly when abundance loss is capped at the maximum model-fitted value (fig. S5), when lower-quality estimates of crop distribution are dropped (fig. S6), when Chao-estimated species richness is used in place of total abundance in the models and projections (figs. S15 and S16), or when projections are based on only bees as a key pollinating taxon (fig. S17).

**Fig. 4.**
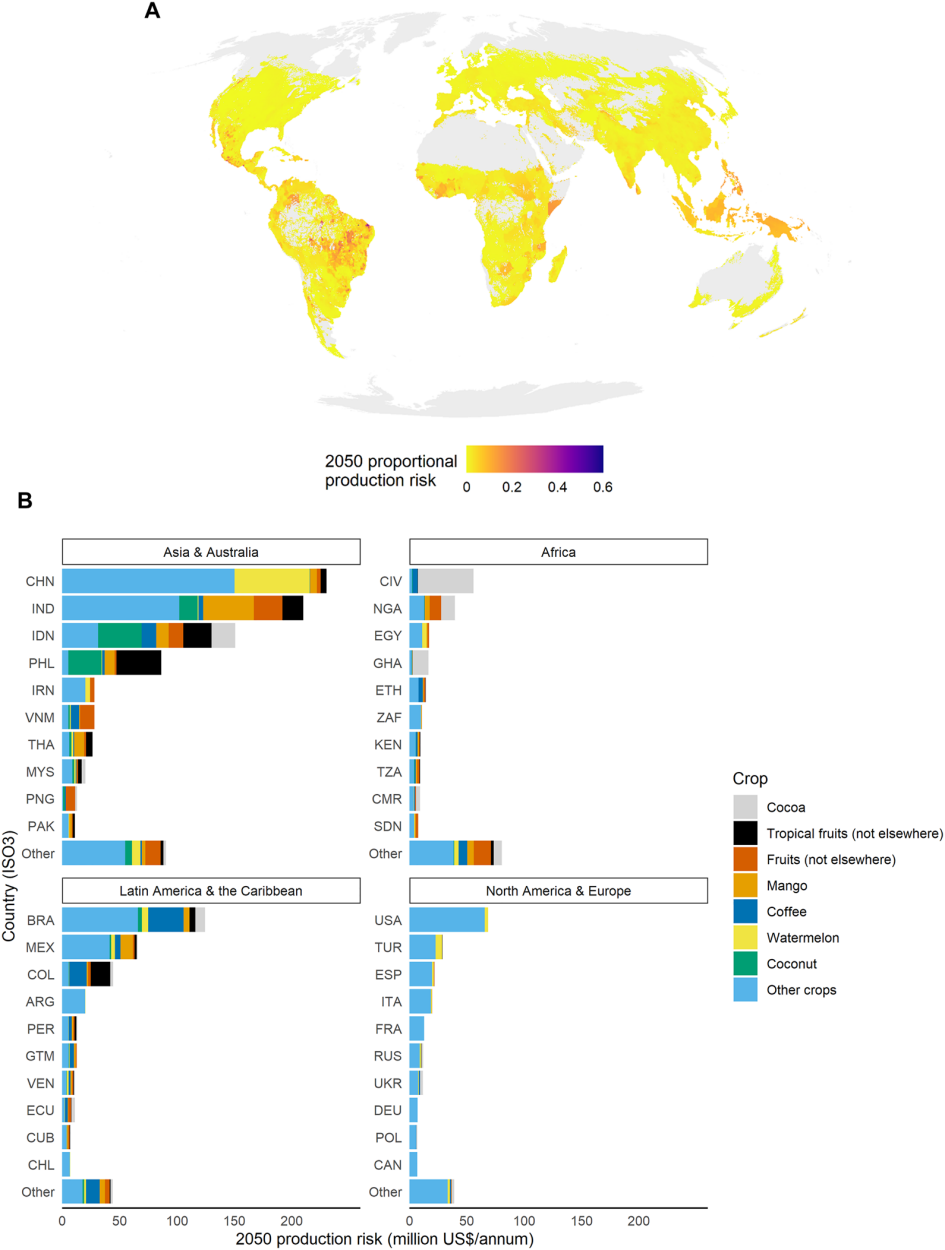
Projected change globally in crop production estimated to be at risk in 2050 under the RCP 6.0 climate scenario, assuming a linear relationship between insect pollinator abundance loss and production loss for crops dependent on animal pollination. All projections are based on mean projections of the standardized temperature anomaly based on temperature estimates from an ensemble of individual climate models. (**A**) The sum of crop production at risk across all crops with some dependence on animal pollination, as a proportion of the production of all crops grown in a location (“proportional production risk”). (**B**) The total crop production at risk for the seven crops with the highest total pollination-dependent production value globally (see fig. S11 for the top 20 crops by pollination-dependent production alone, fig. S12 for country level proportional production risk, and fig. S13 for crop level proportional production risk), in million US$ per annum, broken down into four main geographic regions. Each colored bar represents a pollination-dependent crop group: gray, cocoa; black, tropical fruits (not recorded elsewhere); red, fruits (not recorded elsewhere); orange, mango; dark blue, coffee; yellow, watermelon; green, coconut; and other crops, light blue. Per-tonne values of each crop are for the years 2015–2019 (US$ in 2015–2019 values) taken from (*91*) and total pollination dependent production according to (*47*) and (*36*). Each country is indicated according to its ISO3 code.

Projected risk to crop production in 2050 from insect pollinator abundance losses, as a proportion of all production in a given location, is highest in the tropical regions of sub-Saharan Africa, South America, and Southeast Asia (Fig. 4A; see fig. S8 for individual maps of pollination-dependent crop production and the standardized temperature anomaly). In terms of total production potentially at risk, China, India, Indonesia, Brazil, and the Philippines emerge as being most at risk (Fig. 4B). Among crops, cocoa is estimated to be at highest risk, by a large margin, especially in Africa, followed by mango (particularly in India) and watermelon (notably in China; Fig. 4B). The risk to cocoa production is particularly notable in light of the social and economic context, as most cocoa is produced on small farms (2 to 4 ha) that provide income to between 40 million and 50 million people globally (*58*). Coffee is also expected to have a combination of relatively high production risk (Fig. 4B) and high value, suggesting that regions in which it is grown may experience economic difficulties, unless the pollination service can be replaced cost effectively. Similarly to cocoa, coffee production provides income to millions of small-scale farmers and their families in the tropics (*59*). Therefore, the increased production risk due to loss of pollinators could lead to increased income insecurity for some of the most vulnerable people globally. Our projections of local crop production risk are sensitive to the assumed abundance-production relationship (fig. S9), with the exception of Southeast Asia, which is consistently projected to have high risk, and the temperate realm, which is consistently projected to have low risk (fig. S10).

It is impossible to predict exactly how our estimates of production risk measure will translate into actual crop production losses. There are multiple uncertainties associated with predicting pollinator biodiversity changes and how this affects crop production, some of which we explore here (e.g., the relationship between pollinator abundance and crop production), but many of which we do not or cannot [e.g., the changing distribution of crops (*60*), the economic viability of hand pollination (*61*), the buffering effects of managed pollinators (*62*), the effects of climate change alone (*63*), the uncertainty over whether pollinator abundance is more important than other measures of pollinator diversity (*34*, *64*), the buffering or magnifying effects of landscape composition or agroforestry (*65*, *66*), and other technological solutions such as the breeding or engineering of pollinator-independent cultivars] (*67*). As one example, crops in which hand pollination is already widely practiced, particularly apple, tomato, kiwi, oil palm, and vanilla (*61*), will likely be more resilient than our models would predict. Regardless, at the global scale, assuming that our modeled relationships between standardized temperature change and pollinator abundance are genuine and hold into the future and that the distribution of pollination-dependent production does not significantly change, the relative risk we project is likely to be reflected in challenges for crop production. For cocoa, recent research has focused on the direct effects of climate change on crop production (*68*, *69*), often overlooking those that might be pollinator mediated, probably because the direct effects of climate change are easier to capture and because the set of cocoa pollinating taxa other than midges is unknown (*70*). Solutions to direct and pollinator-mediated effects of climate change may differ. For example, shade trees might protect crops from the detrimental effects of extreme temperatures (*68*) but might not for the ceratopogonid fly pollinators on which cocoa pollination depends (*71*). The focus in previous research on direct climate impacts rather than pollinator-mediated effects is a key gap [but see (*72*)] given that some cocoa varieties are limited more by pollination availability then resource availability (*73*).

**Fig. 5.**
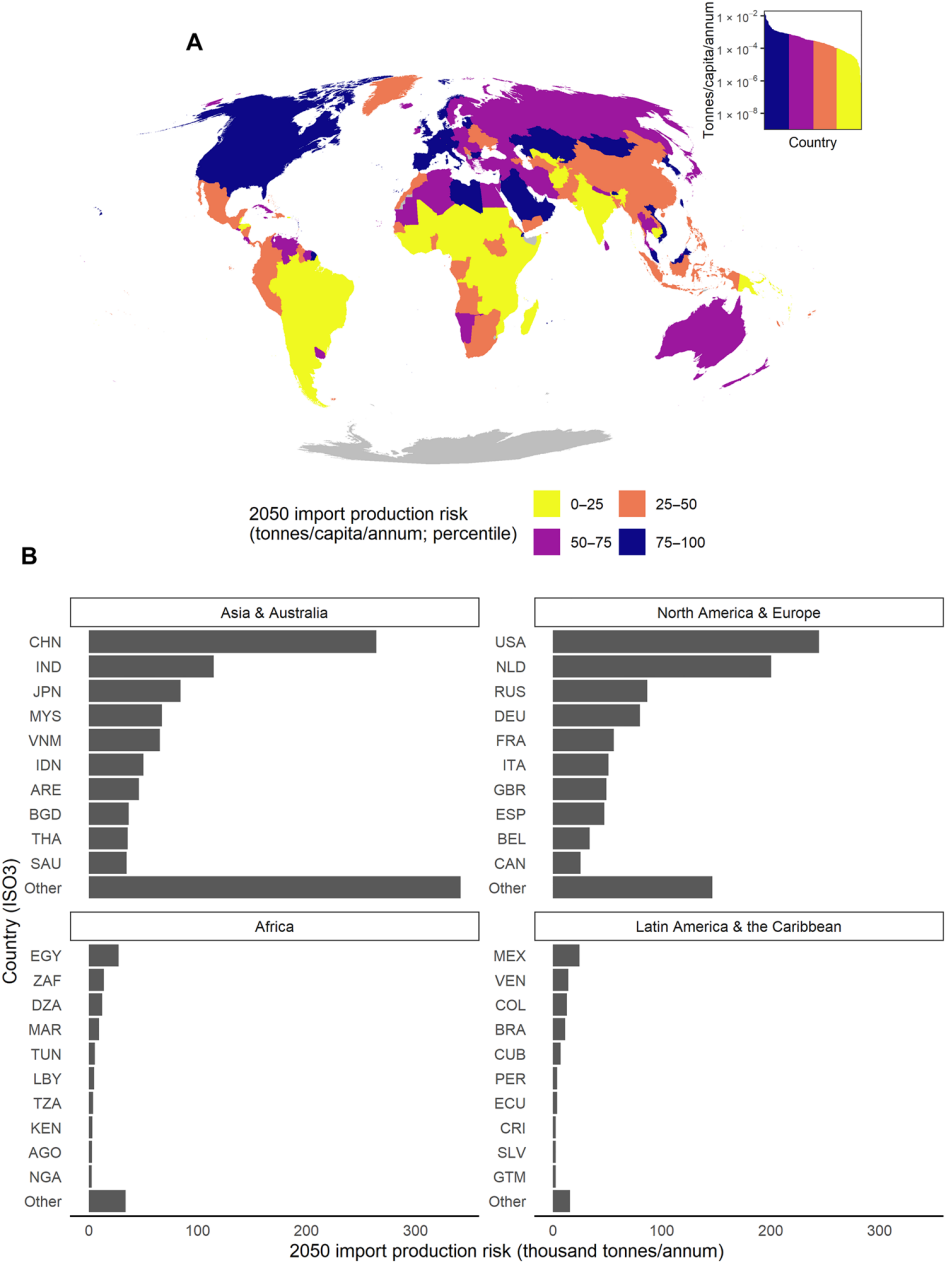
Projected total import risk for 2050 under the RCP 6.0 scenario, assuming a linear relationship between insect pollinator abundance loss and production loss. Projections are based on projected changes in the mean standardized temperature anomaly from an ensemble of individual climate models. Import risk is a measure of how the effects of localized production risk might be distributed among other countries, calculated using trade flow data from (*37*). For each yearly time step into the future, local production risk in each spatial cell is attributed to importers according to the quantity of pollination trade imported from each country. For example, if an importer is dependent on three countries for imports, at a proportion of 30, 50, and 20, then any change in import risk should scale as a function of the local production risk aggregated at those same proportions. (**A**) The geographic distribution of total import risk adjusted for country population size and then converted to a percentile. Colors represent each percentile grouping: yellow, 0 to 25th percentile; orange, 25 to 50th; purple, 50 to 75th; dark blue, 75 to 100th. Inset plot represents the absolute import risk values on a log_10_ scale (although note that the labels are back-transformed), with the same percentile breakpoints. (**B**) The total import crop production at risk in thousand tonnes per annum, for the 10 countries with the highest import production at risk. Each country is indicated according to its ISO3 code. “Other” is the sum of import risk for all other countries in that geographic region.

Countries besides those with high production risk will feel the impact of losses of pollinators and the crops that depend on them through disruption to imports, especially as the most vulnerable crops tend to be valuable export products such as coffee and cocoa. In absolute terms, large countries such as China and the United States have the highest total import risks (Fig. 5B). The Netherlands emerges as having unexpectedly high risk given its size, the third largest overall import risk (Fig. 5B), consistent with its status as the greatest importer and second greatest processor of cocoa beans worldwide (*74*, *75*). Import risk per capita (Fig. 5A) highlights the challenges that could be faced by nations with limited agricultural production capacity, such as many island countries (e.g., Cayman Islands, Aruba, Singapore, the second, third and fourth highest import risk per capita, respectively) or countries with unfavorable environmental conditions for agriculture (e.g., Mongolia, with the 19th highest import risk per capita). Total import risk per capita tends to be high also in northern and high-income countries, particularly continental western Europe, which has large processing industries for crops such as coffee and cocoa. High income and unfavorable environment for agriculture could also account for high import risk per capita for some countries in the Middle East (United Arab Emirates, Kuwait, and Saudi Arabia, which have the 5th, 13th, and 27th highest estimated pollination import risk per capita). Our predictions of import risk are based on trade patterns in the present day (*37*), meaning we do not account for changes in trade flows that will likely occur in the future. Our approach also assumes that all crop production produced in a given country is exported. In other words, we used our trade pattern data to determine how production at risk within a given country should be split among its importers but did not have a value for the proportion of production staying in that country.

Interacting effects of climate change and anthropogenic land use are rapidly restructuring biodiversity (*27*, *76*). Here, we show that the combination of agricultural land use and recent climate change is associated with particularly large reductions in the abundance of insect pollinators. As a result, we predict that the tropics will likely experience the greatest risk of future crop pollination shortfalls, putting at risk the production of crops that depend on insect pollination. Future crop pollination risk is estimated to be highest in areas used to produce cocoa, mango, watermelon, and coffee. Given the many factors that determine crop production and crop price (*77*), the likely effects of insect pollinator losses on crop production are unclear, and even if they do occur, conclusive attribution is likely to be challenging. Such complications likely, in part, explain why identifying a strong effect of pollinator losses on global crop yield and price has thus far been so difficult (*35*, *78*, *79*). Regardless, there is sufficient evidence to suggest that declining insect pollinator abundance will influence crop production risk, particularly for those in the global south (*80*). Such risk could manifest in the form of direct and immediate losses to crop production through pollinator shortfall, fluctuations over time in the stability of production (*81*), or decreased resilience to changes that will happen in conjunction (e.g., the effects of extreme temperature and drought on crop growth). Given the likely buffering effects of landscape composition (*27*, *65*), greater surrounding natural habitat may help mitigate these risks. The health, well-being, and livelihoods of a high proportion of the global population, from small farmers to consumers, rely, to some extent, on the availability and affordability of crops dependent on animal pollination, which is likely to be put at greater risk as a result of future pollinator losses as land use and climate changes intensify.

## MATERIALS AND METHODS

### Biodiversity data for pollinators and nonpollinators

We used the PREDICTS database (*45*, *82*) and a dataset that identifies species from within PREDICTS likely to pollinate plants [see (*17*) for details] to model the response of local pollinator abundance to the interactive effect of climate change and agricultural land use. PREDICTS is a global database of local biodiversity records collected from the primary literature, with a hierarchical structure such that each record is nested into a series of levels [“Source,” “Study,” “Block,” and “Site” (*45*)]. “Sources” represent the individual publications (mostly scientific papers) from which the data were sourced. Sources are divided into separate “Studies” if different sampling methods were used or if the data spanned a very large geographical area (e.g., multiple countries). The locations sampled in each Study are divided into “Blocks” if they form distinct spatial clusters. Last, “Sites” are the locations at which biodiversity was sampled, with the records consisting of a list of named taxa, along with some measurement (most often abundance, sometimes presence or absence, and occasionally an aggregate measure of biodiversity such as species richness). Each record in the PREDICTS database is associated with a land use type (primary vegetation, mature secondary vegetation, intermediate secondary vegetation, young secondary vegetation, plantation, pasture, cropland, and urban), meaning change in biodiversity can be modeled as a function of anthropogenic land use disturbance [e.g., (*17*, *83*)]. Land use types were assigned according to site-level descriptions in the paper from which the biodiversity data was drawn, meaning it will be of a fine scale. Here, we describe only those land use types that are relevant to this study. “Primary vegetation” describes natural habitat with no record of having been destroyed in the past. “Cropland” is an agricultural land use type consisting of herbaceous crops. Each land use type is also categorized according to a land use intensity in PREDICTS, but we do not use that here. Full details of the scheme for classifying land use can be found in (*82*). We focus in this study on the PREDICTS data for insect species. PREDICTS analyses use a space-for-time framework. In other words, biodiversity is sampled across space at varying levels of standardized temperature anomaly, for both cropland and primary vegetation sites, and then we assume that model-inferred spatial differences will be indicative of change over time.

We identified pollinating species in PREDICTS through a semiautomatic approach combining text mining, manual inspection, and expert consultation [see (*17*, *84*) for a detailed description]. We first used the stemmed term “pollinat*” on Scopus to search all abstracts for English language primary research papers. From this set of abstracts, we then used a set of name-entity recognition algorithms to extract all animal species Latin binomial names (*84*). For each animal genus returned by the name-entity recognition algorithms, we then read the corresponding abstracts searching for evidence confirming that genus as pollinating. From this initial abstract scrape, we identified 1013 possible pollinating genera across 3974 abstracts. We considered a pollinator to be an animal for which there is experimental evidence confirming pollination, evidence of pollen carrying, evidence of nectar/pollen feeding, or evidence of nondestructive/nonpredatory flower visitation. Given that the set of pollinators identified from Scopus abstracts could only ever be a sample, we then additionally searched for evidence of pollination across higher-level taxonomic groups (*17*). Specifically, from the confirmed pollinators in our original list of genera, we identified all unique families with at least one pollinator. For each family, we assessed the breadth of evidence for pollination through consulting the abstracts and taxonomic group reference books. For any family with evidence of pollination across multiple branches of that family and no evidence of any species definitely not pollinating, we assumed that the whole family is pollinating. After compiling our list of pollinators from automated text analysis and manual searching, we then consulted a group of seven expert pollination ecologists (O. Adedoja, S. Gavini, E. Kioko, M. Kuhlmann, Z.-X. Ren, and M. Saunders) and removed or added any groups at their suggestion (*17*).

Our data preparation process resulted in two datasets with different subsets of the original set of species in PREDICTS: pollinating insects and nonpollinating insects (see figs. S18 and S19 for the breakdown of the insect pollinators and fig. S23 for the number of records). Our set of nonpollinating species is not strictly a set of confirmed nonpollinators, rather a set of species not confirmed as being pollinators. For each of the two data subsets, we calculated site-level total abundance (the sum of sampled abundances for all species recorded at a site) and estimated species richness [calculated using the Chao estimator (*44*)]. Where sampling effort varied among the sites within a single study (8.9% of records in our pollinating insects dataset and 9.6% in the nonpollinating insects dataset), we divided the abundance values for each measurement by the relative sampling effort at each site, rescaled to a maximum value of 1 within each study, as in (*83*).

### Climate change estimates

We used the Climatic Research Unit Time Series (CRU TS) version 4.03 (*46*) mean daily temperature estimates per month, at a spatial resolution of 0.5° (approximately 55 km at the equator), to calculate a global standardized temperature anomaly for the year of each PREDICTS sample using an approach developed previously (*27*). Although extreme temperatures have been shown to predict contemporary changes in biodiversity better than mean temperatures (*85*), we used the latter here since they provide a measure of the central tendency of change in temperature per month. Mean temperatures have been used in a similar manner for a number of other studies on insect thermal tolerance [e.g., (*28*, *86*)], in which they have been shown to be informative of insect biodiversity change. To calculate our standardized temperature anomaly, we first calculated a 30-year baseline temperature for the years 1901–1930 as the mean temperature across all 360 monthly mean daily temperatures for each cell. For each PREDICTS site, we then calculated contemporary temperature as the mean temperature across the 12 months up until the last month of sampling at that site. We then calculated a temperature anomaly for each site as the difference between the baseline and contemporary average temperatures. We then standardized this climate anomaly by dividing the anomaly at each site by the SD across the 360 monthly mean daily temperatures in the baseline period. A standardized temperature anomaly of less than 0 indicates a region that has cooled since the baseline. A value between 0 and 1 indicates a region that has warmed, but current average temperature remains within 1 SD of the variability in baseline temperatures. A value greater than 1 indicates a region in which average warming is 1 SD greater than the variability in the baseline (i.e., it is now experiencing high novel temperatures). We also calculated a global map of the standardized temperature anomaly for the period 2004–2006, also using the CRU TS version 4.03 (fig. S8). The period 2004–2006 coincides with the midpoint of sampling in the PREDICTS database (*45*).

### Biodiversity responses

To model the interactive effects of land use and recent climate change on pollinator (and nonpollinator) abundance, we built linear mixed-effects models predicting total abundance as a function of land use type (primary vegetation and cropland), standardized temperature anomaly, and their interaction (see the spatial distribution of sites in fig. S14, the values of abundance in fig. S20, the length of sampling period in fig. S21, the number of sites in table S1, and the number of species in table S2). We did not use a generalized linear model with Poisson errors because most recorded measurements are noninteger values. Pollinator abundances are, in some cases, noninteger values after site level sampling effort has been accounted for or if abundance was recorded as a density or relative abundance. We focused on primary vegetation and cropland given our interest in assessing how pollinator biodiversity change may affect crop production that depends on animal pollination. We didn’t separate cropland or primary vegetation sites by their intensity of use because there are relatively few data for pollinator species compared to insects as a whole and because the distinction between land use types is more important than between levels of agricultural intensity, for understanding the impact of land use–climate interactions (*27*). We log_e_-transformed all total abundance values (adding one because of zero values) to normalize the model residuals. Because of the nested nature of the database (*45*), we considered a random intercept of study identity to account for variation in sampling methods, sampling effort, and broad geographical differences among studies, and a random intercept of spatial block within study to account for the spatial structuring of sites. Models that included this random-effects structure had a lower Akaike information criterion (AIC) value than models with a simpler combination of the same random effects (i.e., study identity only). Given the hierarchical structure of the PREDICTS database, with a large amount of the variation in recorded biodiversity being explained by the random effects (especially study identity), typically the conditional R squared is very high and the marginal R is squared very low (see table S3) (*15*). To infer the interactive effect of the standardized temperature anomaly and land use type, PREDICTS models require variation in land use type (i.e., cropland or primary vegetation) within studies.

### Potential future risk to crop pollination

We used our model of insect pollinator abundance, combined with information on crop dependence on animal pollination and projections of future climatic changes to predict geographic (change in space) and temporal (change in time) patterns of potential risk to crop pollination. We focus on three forms of risk: total production risk, proportional production risk, and import risk. Production risk is a measure of the total crop production potentially at risk of pollination shortfalls. Proportional production risk is a measure of the crop production at risk as a proportion of the total production for a given cell, crop, or country. Import risk is a measure of risk to imports of crops dependent on animal pollination via international trade. We focus in this study on insects, which make a particularly large contribution to crop pollination (*36*).

Our projections are based on the assumption that a projected loss in pollinator abundance will be associated with an increased risk to crop production from loss of pollination services. There is strong evidence linking insect abundance with crop pollination (*33*, *42*). Furthermore, even if pollinator losses don’t affect crop yields directly, they may reduce the resilience of crop production in the face of other environmental changes (*39*). Nevertheless, there are three core areas of uncertainty. First, we do not know if there is a mechanistic link between the interactive effects of land use and climate change on pollinator abundance. We reason that a significant interactive effect is at least likely, however, given prior localized studies demonstrating a synergistic effect of climate change and anthropogenic land use in insects (*76*, *87*). Second, we do not account for changes in the distribution of crops over time, which may occur because of direct effects of climate change, indirect feedbacks caused by pollinator losses, or other environmental or socioeconomic factors. Therefore, our projections should be seen as estimates of the risk posed to crops where they are currently grown, which is still an important consideration for food security and livelihoods. Third, it remains unclear exactly how local abundance change will affect crop pollination, how abundance change will interact with richness change, and, in turn, how crop pollination will relate to yield change. We account for this uncertainty as much as possible by testing alternative possible relationships between pollinator abundance and production risk (see below for details). We also include a supplementary analysis in which we model species richness [as an alternative biodiversity metric with a potential link to pollination provision (*42*)] as a function of the interactive effects of climate change and land use and project crop pollination risk using these models (figs. S15 and S16). We also develop a separate set of models and projections of pollination risk based only on bee species.

Here, we opted to focus on abundance for a few reasons. First, the relative contributions of different biodiversity metrics are not clear, precluding the calculation of an indicator of crop pollination risk that aggregates multiple biodiversity metrics. Furthermore, with a single metric, we can vary the slope of its relationship with crop production, but aggregating metrics, we would have to iterate over a set of plausible weightings and then, for each of those plausible weightings, vary the slope of its relationship to production. Second, the mechanistic link between pollinator abundance, pollen load, pollen deposition, and crop pollination is an intuitive causal pathway. For some combination of metrics or metrics other than abundance, the logic of this causal pathway falls away, such that we lose a framework in which we can vary the relationship of these parameters to each other. In reality, however, true risk will emerge as a function of multiple different facets of biodiversity, with some links as yet poorly understood. Given all the uncertainties, our projections should be interpreted as a measure of relative risk to crop production rather than projections of absolute yield loss.

We first used the EarthStat global maps of the production of individual crops (*47*), in combination with the animal pollination dependencies reported in (*36*), to build a map of global crop production dependent on animal pollination for the year 2000. The spatial resolution of each crop in EarthStat is 0.08°, equivalent to ~10 km at the equator. For each crop represented in EarthStat, we adjusted total production according to the proportional dependence on animal pollination, as reported in a literature review of the primary literature in (*36*) (essential = 0.95; great = 0.65; modest/great = 0.45; modest = 0.25; little = 0.05; no increase = 0). Some crop groups in EarthStat are represented by multiple individual crops in (*36*). For these groups, we calculated the mean dependence on animal pollination (see table S4). We exclusively used (*36*) for pollination dependence ratios for internal consistency or, in other words, for a set of ratios that we could be confident were meaningful relative to each other. We then summed the dependence-adjusted production values for all crops (*N_c_*) grown in each cell as

$${\mathrm{PollinationProd}}_{i}=\sum_{c=1}^{c=N_{c}}{\mathrm{Prod}}_{ci}d_{c}$$

where PollinationProd*_i_* is the total crop production in metric tonnes that is dependent on animal pollination in cell *i*, Prod*_ci_* is the total production of crop group *c* in cell *i*, and *d_c_* is the proportional dependence of crop *c* on animal pollination (see fig. S11 for the total production dependent on animal pollination for the top 20 crops and fig. S8 for the global distribution of all animal pollination–dependent production). We also calculated the total production of all crops in any given cell (regardless of dependence on animal pollination) as

$${\mathrm{TotalProd}}_{i}=\sum_{c=1}^{c=N_{c}}{\mathrm{Prod}}_{ci}$$

where TotalProd*_i_* is the total production of all crops in cell *i*. Although our study was focused on insects, we considered all crop groups with maps in EarthStat because none of these crop groups relies exclusively on pollination by vertebrates of noninsect invertebrates.

To project future risk to crop production, we used the ISIMIP (Inter-Sectoral Impact Model Intercomparison Project) predicted temperature anomalies from (*88*) to calculate the standardized temperature anomaly for each year between 2016 and 2050, under three RCP scenarios (2.6, 6.0, and 8.5; in the main text, we present results only for RCPs 2.6 and 6.0) using an ensemble mean of the general circulation climate models GFDL, HadGEM2, IPSL, and MIROC5. RCP 8.5 represents a worst-case high-emissions scenario, 6.0, a pathway with some degree of mitigation, and 2.6, a pathway with significant reductions in emissions (*89*). RCP 2.6 has a multimodel median–predicted increase of 1.5°C in global average temperatures by 2100 compared to the preindustrial climate; RCP 6.0 has a predicted increase of 3°C, and RCP 8.5 has a predicted increase of 4.9°C (*48*). ISIMIP temperature anomalies were added to average monthly temperatures across a historical baseline period of 1979–2013. We used the annual standardized temperature anomaly estimates to calculate a 3-year rolling average to smooth change in risk over time. For each 3-year projection window, insect pollinator abundance on cropland was predicted according to the model in Fig. 1 (left) for all cells containing crop production dependent on animal pollination (note that our predictions of risk are made at the finer scale of the crop production data, rather than the coarser resolution of the climate data). These abundance values were then expressed as the proportional loss of abundance compared to the abundance expected on croplands that have experienced no warming (i.e., standardized temperature anomaly of 0) as

$$l_{ti}=1-\frac{a_{ti}}{a0_{i}}$$

where *l_ti_* is the projected abundance loss for (3-year) time period *t* and cell *i*, *a_ti_* is the model-estimated abundance on cropland in cell *i* under projected warming for time period *t*, and *a0* is the model-estimated abundance on cropland in cell *i* under no warming (standardized temperature anomaly = 0). In each cell, animal pollination–dependent crop production was then adjusted for the percentage reduction in abundance at that time step before summing production at risk for all cells (*N_i_*) as

$${\mathrm{ProdRisk}}_{t}=\sum_{i=1}^{i=N_{i}}{\mathrm{PollinationProd}}_{i}l_{ti}$$

where ProdRisk*_t_* is the total crop production at risk in time period *t*.

We carried out a set of robustness checks for our total production risk projections. First, we checked for the influence of single climate models on our projections. Specifically, we calculated one projection as the average of all models for that RCP scenario and a set of additional jack-knifed projections, dropping each climate model in turn (fig. S4). Second, for the ensemble average of all climate models and for RCPs 2.6, 6.0, and 8.5, we checked the extent to which extrapolating abundance loss beyond the lowest fitted abundance value in the models of land use and climate change impacts (i.e., the greatest model-estimated abundance loss) affected our projections (fig. S5). Third, we checked the extent to which our projections were robust to differences in the data quality in the EarthStat crop production maps by iteratively filtering the production estimates to include only higher-quality estimates (fig. S6). EarthStat crop production data are broken down into 5 quality levels (1, 0.75, 0.5, 0.25, and 0), from highest to lowest data quality: 1, county level census data; 0.75c state level census data; 0.5, census interpolated data, from within 2° latitude/longitude; 0.25, country level census data; and 0, missing census data. Fourth, we used two different approaches to test whether our projections are influenced by considering the fact that insects are only active in some months of each year (in our main projections, we included all months of the year in the calculation of the standardized temperature anomaly). The first approach followed the methodology of (*27*), defining active months as those that have an average temperature of at least 10°C in the 5 years before each PREDICTS sample and then calculating the standardized temperature anomaly using only those months. The second approach defined active months based on temperatures in the baseline period, i.e., calculating the standardized temperature anomaly based only on months for which the average temperature between 1901 and 1930 was at least 10°C. For both of these approaches, we plotted the mean total abundance change (for just the pollinating insects) on cropland relative to sites that have not changed, for a set of potential scenarios (i.e., fig. S3). Fifth, we built additional models to test whether abundance responses to the interactive effects of climate change and land use differed among taxonomic groups. In the first approach, we built a separate model for each of the main pollinating insect orders (fig. S1), and in the second, to test whether our results are overly influenced by individual taxa, we built a series of models dropping each pollinating insect family in turn (fig. S2). Sixth, we built an analogous model in which we fit bee abundance alone as a function of the interaction between land use type and standardized temperature anomaly and then derived a risk map for this model alone (fig. S17). Seventh, we built an analogous model in which we fit species richness [estimated using the Chao estimator (*44*)] as a function of the interaction between land use type and standardized temperature anomaly (fig. S15) and then derived a risk map for this model alone (fig. S16). Eighth, we jack-knifed the predictions for cropland and primary vegetation for the top 10 most common sampling methods (“flight trap,” “light trap,” “pit-fall traps,” “baited traps,” “line/belt transects,” “aerial flight-inception trap,” “live traps,” “sweep net,” “malaise traps,” and “aerial transect”) used in the original biodiversity sampling (figs. S22 and S23). Ninth, we fit an analogous mixed effects model including an interaction between land use type and mean annual temperature with the original set of fixed effects to test whether including this covariate had an impact on the coefficients (table S6). Tenth, we fit mean annual temperature and the standardized temperature anomaly as main effects for cropland and primary vegetation sites modeled separately (fig. S25; see fig. S24 for the site-level correlation of mean annual temperature and standardized temperature anomaly).

Given the uncertainty in the relationship between pollinator abundance and crop production, we tested a series of potential relationships, including linear, or with some form of concave or convex relationship. In other words, it could be that crop production declines slowly until a large proportion of pollinators have been lost (concave relationships). Alternatively, crop production may decrease rapidly with even small reductions in pollinator abundance (convex relationships). We used two functions, describing concave relationships as

$$c_{ti}=-\sqrt{{\left(l_{ti}-1\right)}^{z}}+1$$

and convex relationships as

$$c_{ti}=\sqrt{{l_{ti}}^{z}}$$

where *c*_*ti*_ = a constant that can be switched for *l*_*ti*_ to give crop production loss at time *t* in cell *i*, and *z* = a constant describing the concavity or convexity of the relationship between local pollinator abundance loss and risk of crop production loss. For both concave and convex relationships, we considered four values of *z* (4, 8, 16, and 32), describing increasingly steep concave or convex relationships. We assumed that all lines meet at the extreme values of pollinator abundance loss (i.e., no risk to crop production where pollinator abundance is equal to or higher than in the natural baseline condition and a proportional risk of 1 where all pollinator abundance is lost). Here, we present only the linear and concave relationships because these seem more likely to describe the true relationships (*55*). In fig. S7, we also present the results based on the convex projections (*54*).

To identify geographic regions in which a high proportion of crop production could be at risk, we expressed pollination risk as a proportion of the total production of all crops within each cell as

$${\mathrm{PropRisk}}_{ti}=\frac{{\mathrm{ProdRisk}}_{ti}}{{\mathrm{TotalProd}}_{i}}$$

where PropRisk*_ti_* is the fraction of all crop production in cell *i* and time period *t* at risk from pollinator biodiversity losses. A value of 1 therefore indicates a hypothetical situation in which all of the crop production in that cell is dependent on animal pollination, and predicted insect pollinator abundance loss is 100% (i.e., one would expect a very high risk of crop production loss from pollination shortfalls).

To investigate which countries and regions of the world might expect the greatest crop pollination risk, we intersected our mapped estimates of production risk with a global map of country borders (*90*). For each country, we calculated the median risk in the year 2050 across grid cells (“overall risk”), and the total change between the start (2016) and end (2050) of the series (“change in risk”).

To estimate the potential financial risk associated with pollinator biodiversity loss, we estimated, for each country, the total value of pollination-dependent crop production per country. Here, we present this total value, while in fig. S12, we present estimates divided by total national gross domestic product (GDP). We calculated the total value of crop production dependent on animal pollination by multiplying the total animal pollination–dependent production for each crop in each country by the estimated per-tonne value of each crop (*91*) and then summing these values across all crops grown in a given country. Per-tonne values for each crop were estimated by calculating the mean producer price of each crop in each country for the years 2015–2019 (US$ in 2015–2019 values) using Food and Agriculture Organization estimates (*91*). For each crop, we then took the median value across countries as a global estimate of the per-tonne value of each crop. These values represent estimations of the price paid to producers at the point of initial sale (*91*). We retrieved estimates of each country’s GDP in millions of US$, from the package “rworldmap” (variable name “GDP_MD_EST”) (*90*). In fig. S13, we present estimates per crop for median risk and change in risk, across all the cells in which that crop exists.

As a last step, we used our measure of local production risk to calculate a measure of overall import risk and change in import risk. We used estimates of the quantity of production (in metric tonnes) dependent on animal pollination (accounting for the fractional dependence of each crop on animal pollination) imported by each country between 2001 and 2015, broken down by country of origin (*37*). The data used here differs from that of (*37*) in that it does not include a measure of average cropland isolation from natural habitat. This adjustment was done so that production and flow calculations would match. Global patterns of import risk detected here are not affected by this calculation change.

To convert the estimates of import flow into a measure of import risk, we reasoned that each unit of production dependent on animal pollination produced in a given country can be attributed to an importer according to the proportion that it imports from that country. To calculate this import risk for 2050, we multiplied the import flow of crops dependent on animal pollination from each producing country (as a proportion of the total imported from all producers) by the total production at risk across all cells in the exporting country in 2050. For each importer, we summed this value across all exporters. Import risk for a given importer country (*I*) at a given time (ImportRisk_t*I*_*)* is therefore calculated as

$${\mathrm{ImportRisk}}_{tI}=\sum_{E=1}^{E=N_{E}}{\mathrm{ProdRisk}}_{tE}P_{E}$$

where *P_E_* is the proportional flow between country *E* and *I* and *N_E_* is the total number of exporters to country *I.* Thus, for example, if one importer receives 20% of one country’s exports, 20% of another, and 10% of a third and each of those countries has a local production risk at time *t* of 100, 200, and 500 tonnes, then ImportRisk*_tI_* will be

$${\mathrm{ImportRisk}}_{tI}=(100\times 0.2)+(200\times 0.2)+(500\times 0.1)$$

We defined this value as the overall import risk, which we then divided by the mean total population size over the period 2015–2019 [sourced from Our World in Data (*92*)] of each country to give a per-capita estimate. All analyses were carried out in R version 4.0.5.

## Supplementary Material

20231012-1
