# Supplementary material for: Key tropical crops at risk from pollinator loss due to climate change and land use
Source: Sci Adv. 2023 Oct 12;9(41):eadh0756. doi: 10.1126/sciadv.adh0756 (PMC10569713; doi:10.1126/sciadv.adh0756)
Supplement: Supplementary file 2 — Figs. S1 to S25 Tables S1 to S6 [file sciadv.adh0756_sm.pdf]

Science Advances
AAAS

Supplementary Materials for

Key tropical crops at risk from pollinator loss due to climate change and land use

Joseph Millard *et al.*

Corresponding author: Joseph Millard, joseph.millard@nhm.ac.uk

Sci. Adv. **9**, eadh0756 (2023)
DOI: 10.1126/sciadv.adh0756

This PDF file includes:

Figs. S1 to S25
Tables S1 to S6

Supplementary Figures

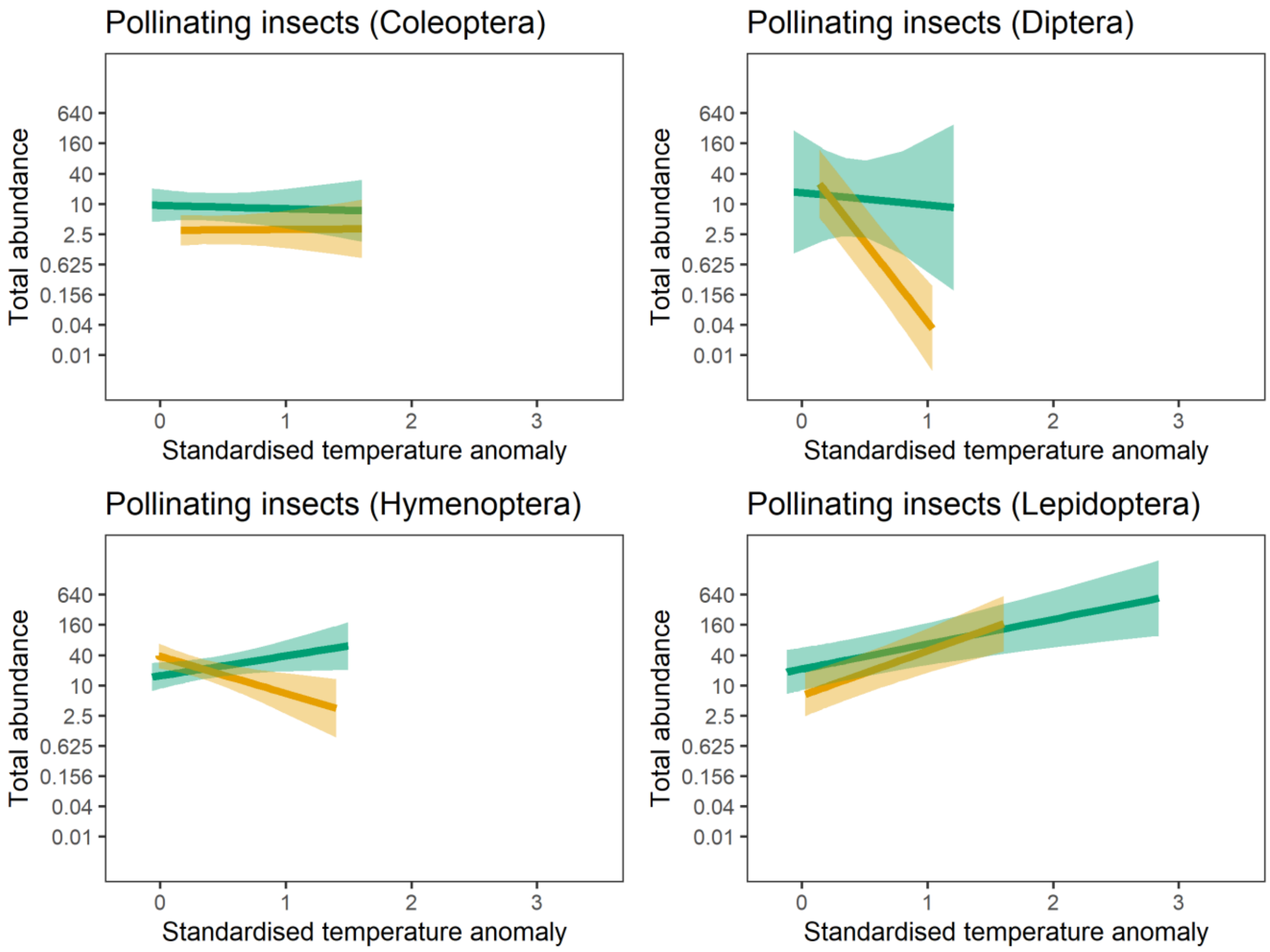

Figure S1. Response of the total abundance of individual orders of pollinating insects to standardised temperature anomaly on primary vegetation and cropland. Each panel represents a linear mixed-effects model for one order (Coleoptera, Diptera, Hymenoptera, and Lepidoptera; note that each taxonomic order has been fit separately rather than as an interaction). Coloured lines represent mean fitted estimates for each interaction, and shading 95% confidence intervals around the mean: green = primary vegetation; orange = cropland. Note that abundance is plotted on a $\log_e$ scale (although the labels are back-transformed).

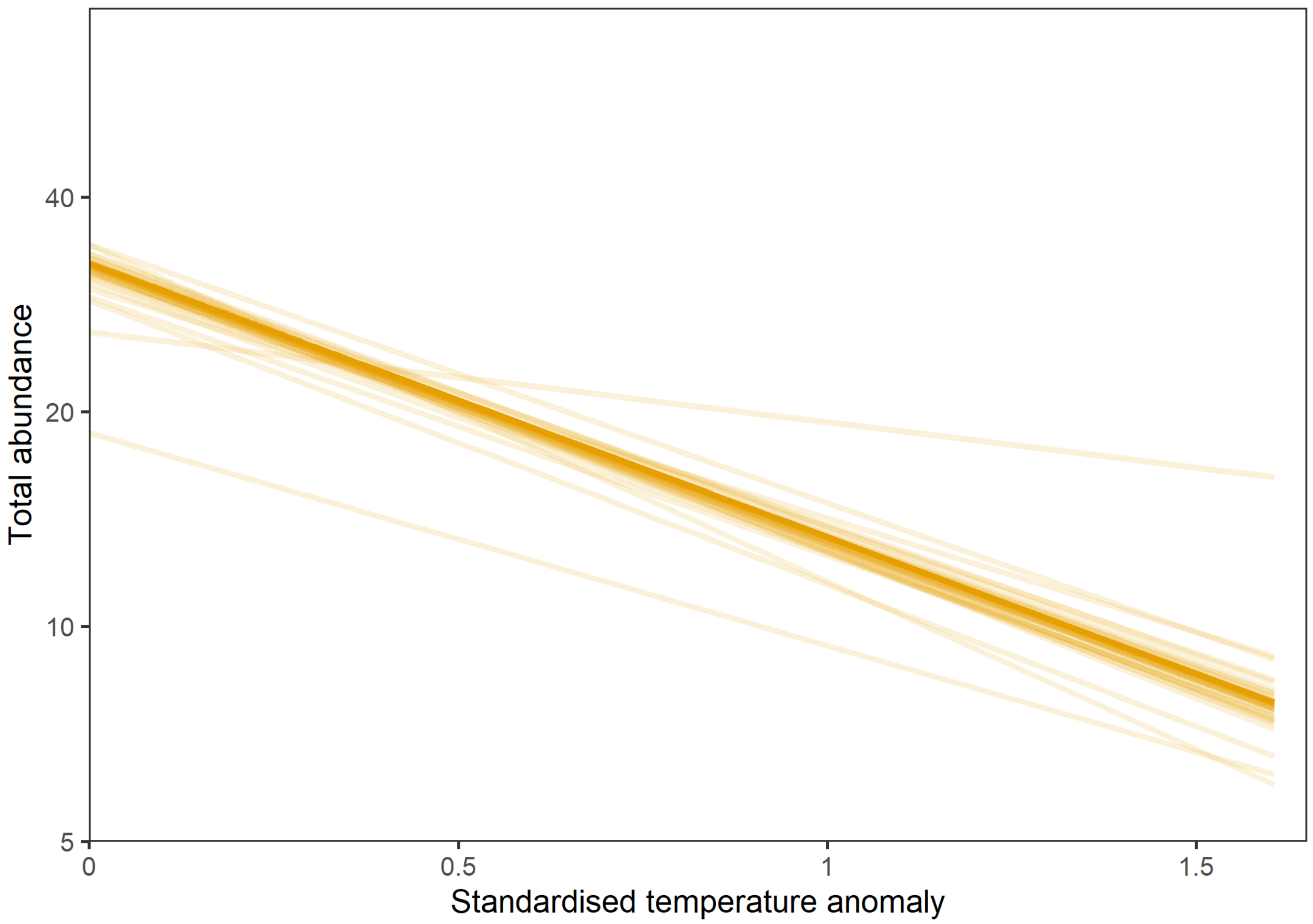

Figure S2. Response of pollinating insect total abundance to the standardised temperature anomaly on cropland, jack-knifed by dropping each taxonomic family in turn. Predictions were derived from a linear mixed-effects model of total abundance as a function of land use in interaction with the standardised temperature anomaly. The dark orange line represents the trend for all insect families combined, and each translucent line shows the modelled relationship with one family excluded. Note that abundance is plotted on a $\log_e$ scale (although the labels are back-transformed).

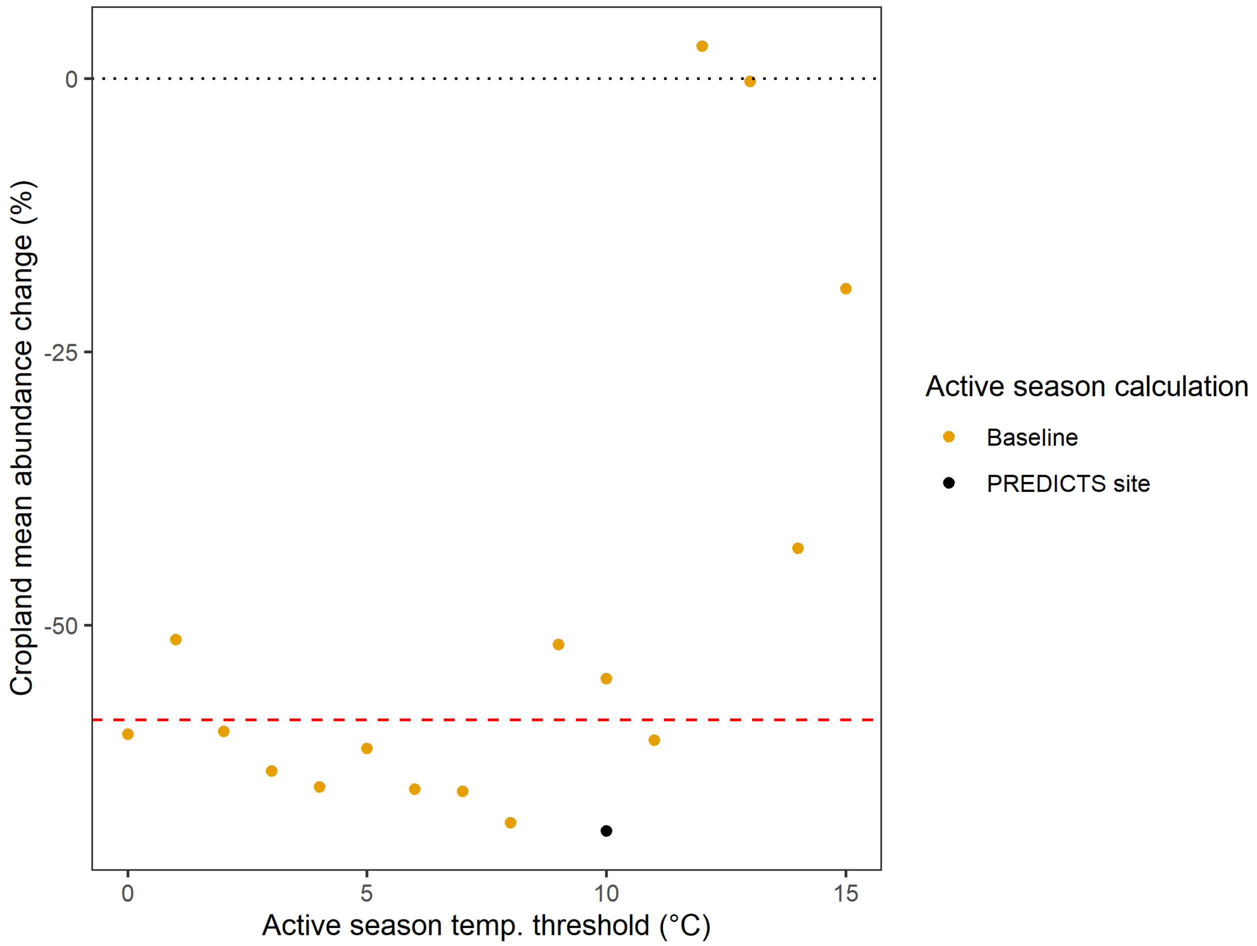

Figure S3. Response of pollinating insect total abundance to standardised temperature anomaly on cropland for a set of active season thresholds, calculated using two approaches: 1) Defining active months as any months in the baseline that have a temperature of at least 10°C, and then calculating the STA for only these months in the period 5 years previous to each PREDICTS site (i.e. 'Baseline' calculation, orange dots); and 2) defining active months as any months 5 years previous to each PREDICTS site that have a temperature of at least 10°C, and then calculating the STA for only this set of months in the baseline, as in the approach used in (*27*)) (i.e. 'PREDICTS site' calculation, black dots; note that this is for only one simulation, since these take much longer to run). Each point represents the percentage change in insect pollinator total abundance on cropland sites, between a standardised temperature anomaly of 0 and 1. The red dashed line represents change predicted on cropland with no active season adjustment, as in all main text figures.

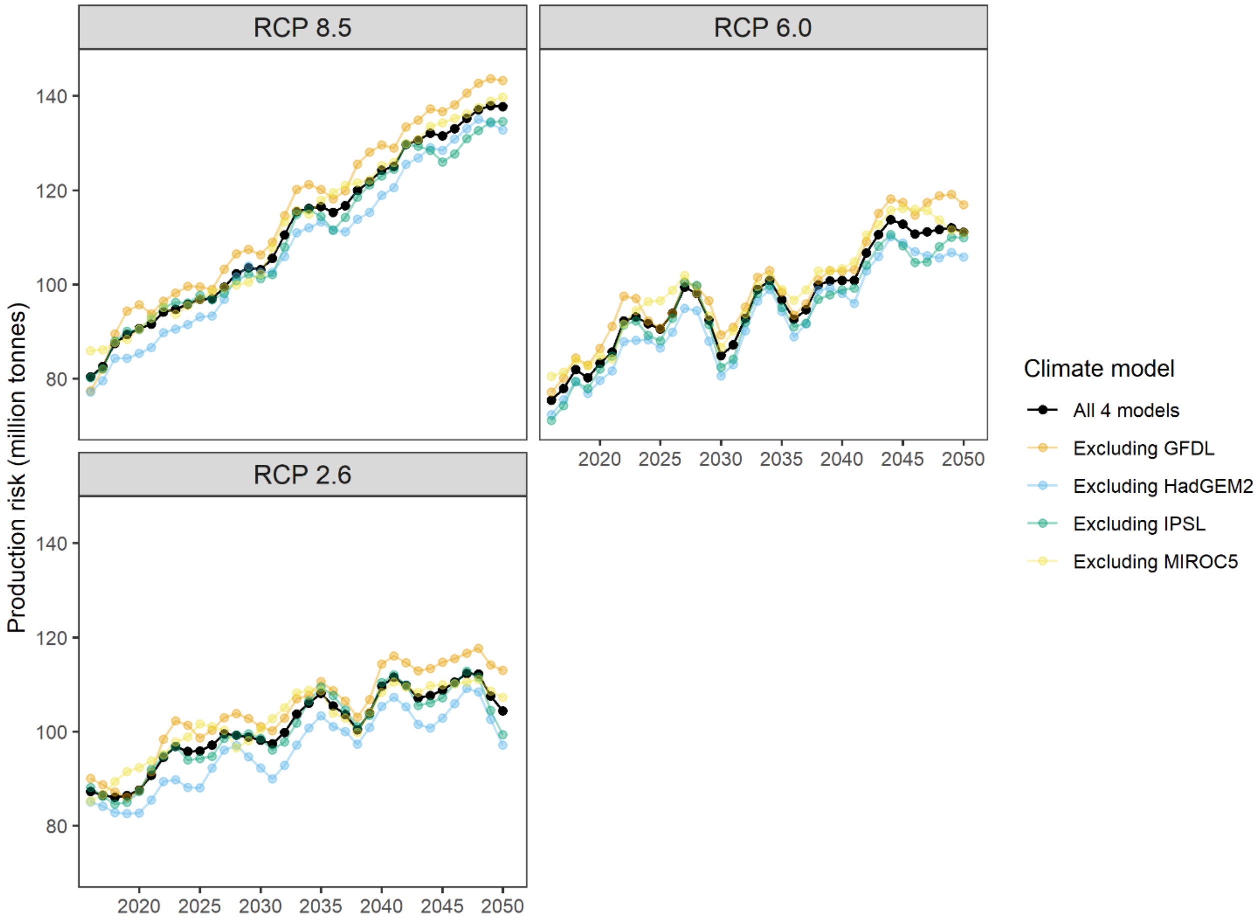

Figure S4. Sensitivity of projected changes in total production risk to dropping temperature projections from individual climate models. Shown are projections for three different RCP climate scenarios (8.5, 6.0, and 2.6), assuming a linear relationship between abundance loss and production risk, either as the average across all general circulation climate models, or dropping each climate model in turn (GFDL, HadGEM2, IPSL, and MIROC5).

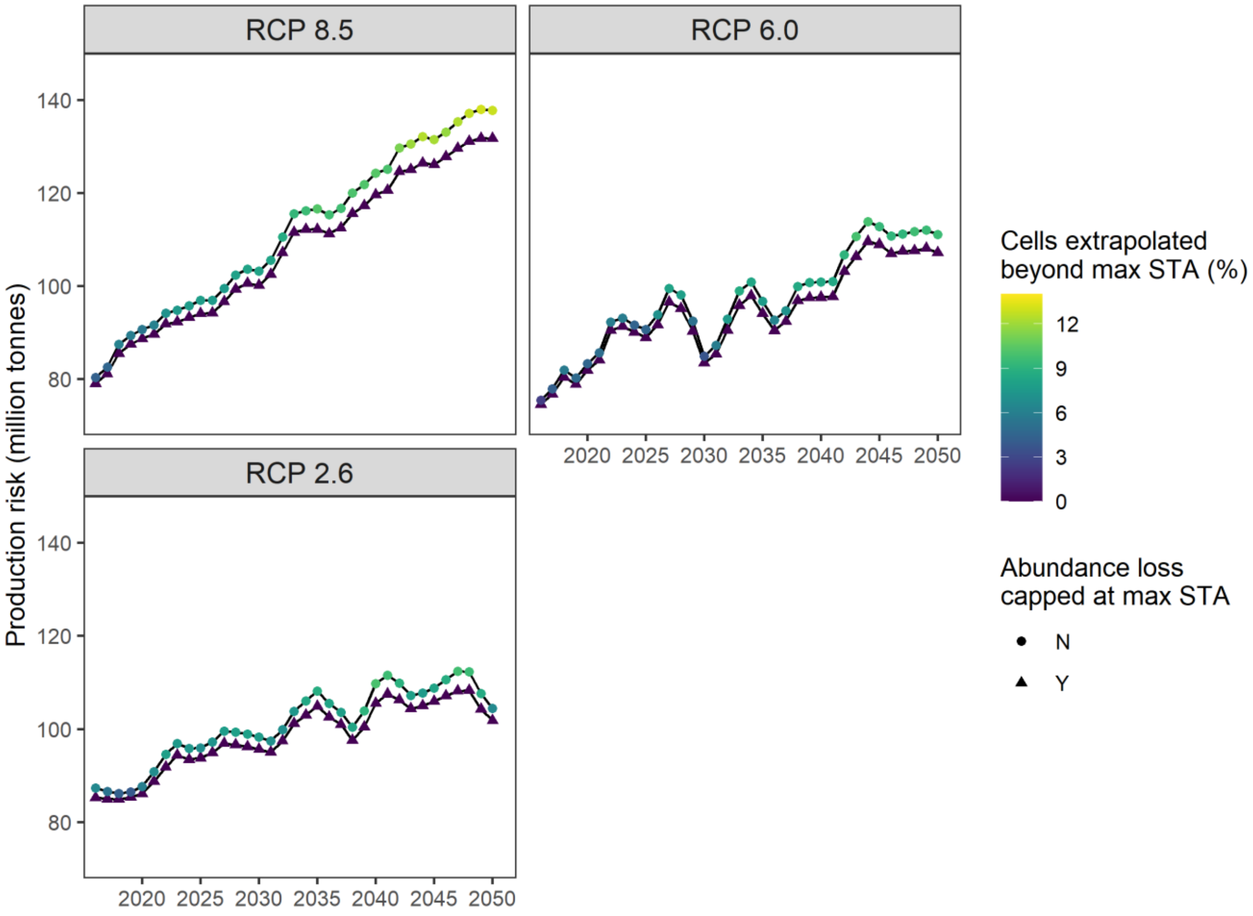

Figure S5. Sensitivity of projections of risk to crop production from extrapolating the underlying models beyond the range of sampled values of recent climate change. Shown are projected changes in total production risk under three RCP scenarios (8.5, 6.0, and 2.6), using average projected temperature changes from four climate models (GFDL, HadHEM2, IPSL, and MIROC5), assuming a linear relationship between abundance loss and production risk. Circles represent a projection in which abundance loss is extrapolated beyond the maximum standardised temperature anomaly (STA) (as in Figure 2 in the main text), whereas triangles represent a projection in which abundance loss is capped at the maximum sampled STA value of 1.61. Colours indicate the percentage of cells at each time step that have been extrapolated beyond the maximum STA in the non-capped projections.

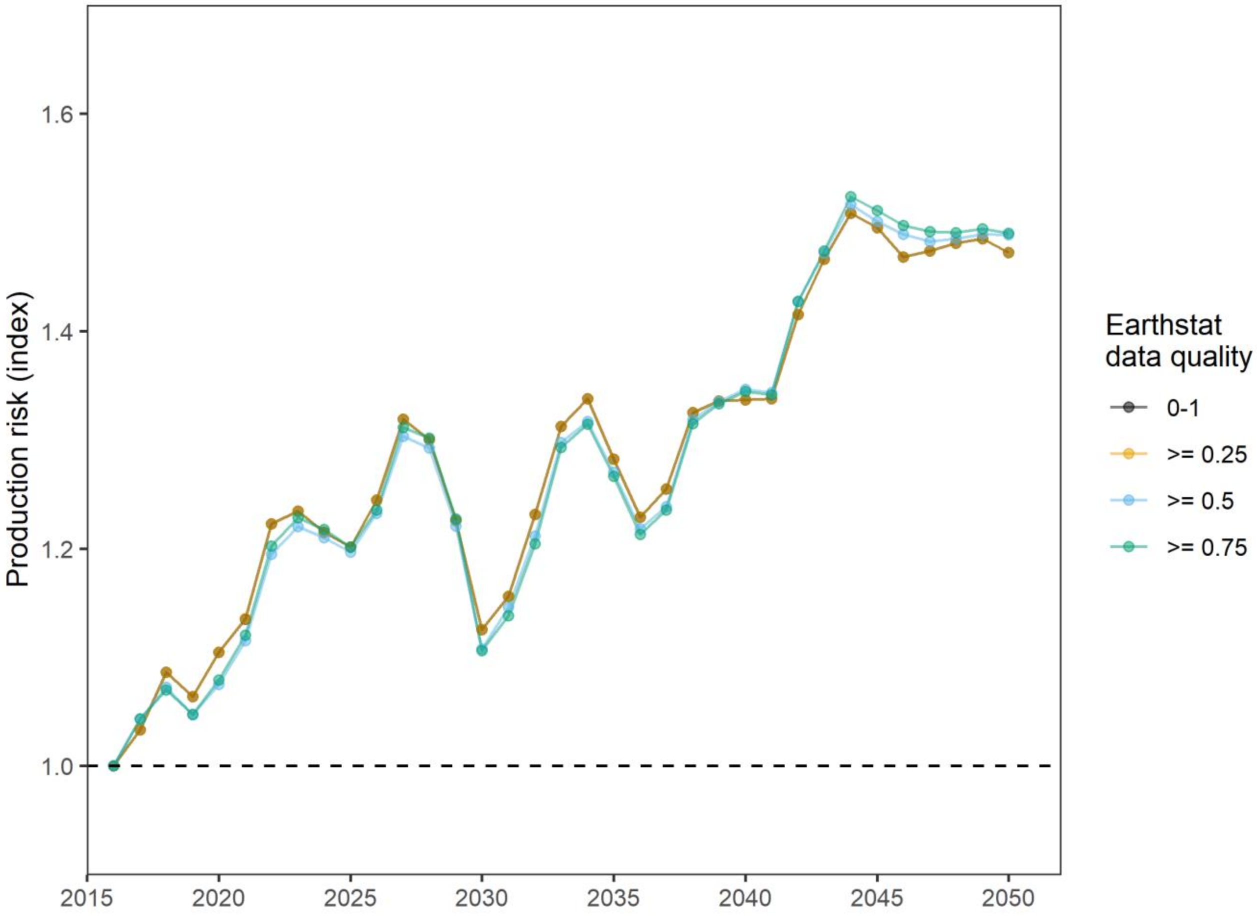

Figure S6. Sensitivity of the projected index of relative production risk to variation in the quality of mapped estimates of crop production. Projections are shown for the RCP 6.0 climate scenario, with temperature projections based on the average of four climate models (GFDL, HadHEM2, IPSL, and MIROC5), assuming a linear relationship between abundance loss and production risk. Colours represent projections based on crop-production estimates filtered according to different levels of reported data quality (*47*): 1: county-level census data; 0.75: state-level census data; 0.5: regional interpolation from census data; 0.25: country-level census data; 0: no census data.

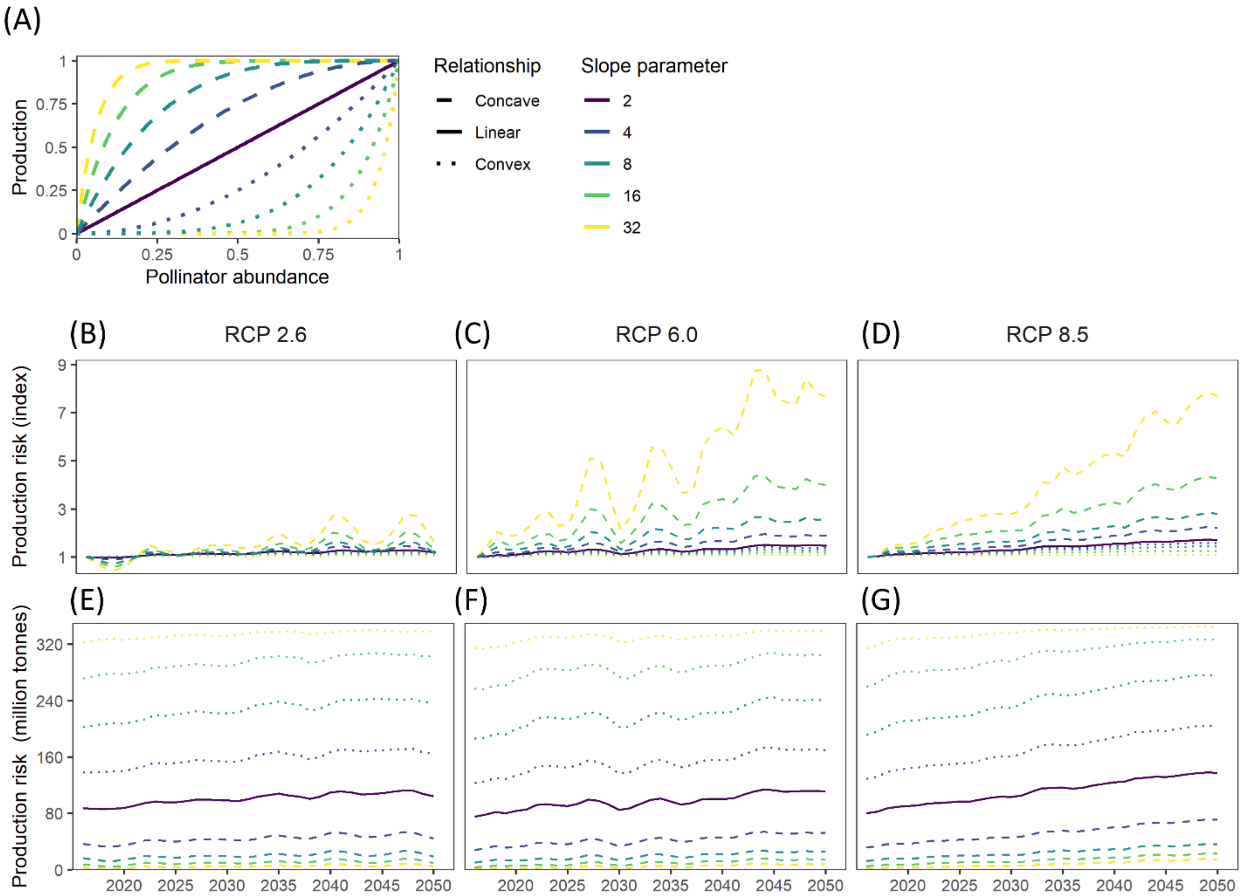

Figure S7. Sensitivity of projected crop production risk to the assumed relationship between pollinator abundance and production risk. Assumed relationships (shown in A) were linear (slope parameter = 2), as well as concave (dashed lines) and convex (dotted lines) relationships of increasing steepness (slope parameters between 4 and 32). Shown are projections of both the relative index of risk (B-D) and total production risk (E-G), for three RCP climate scenarios (2.6: B, E; 6.0: C, F; and 8.5: D, G).

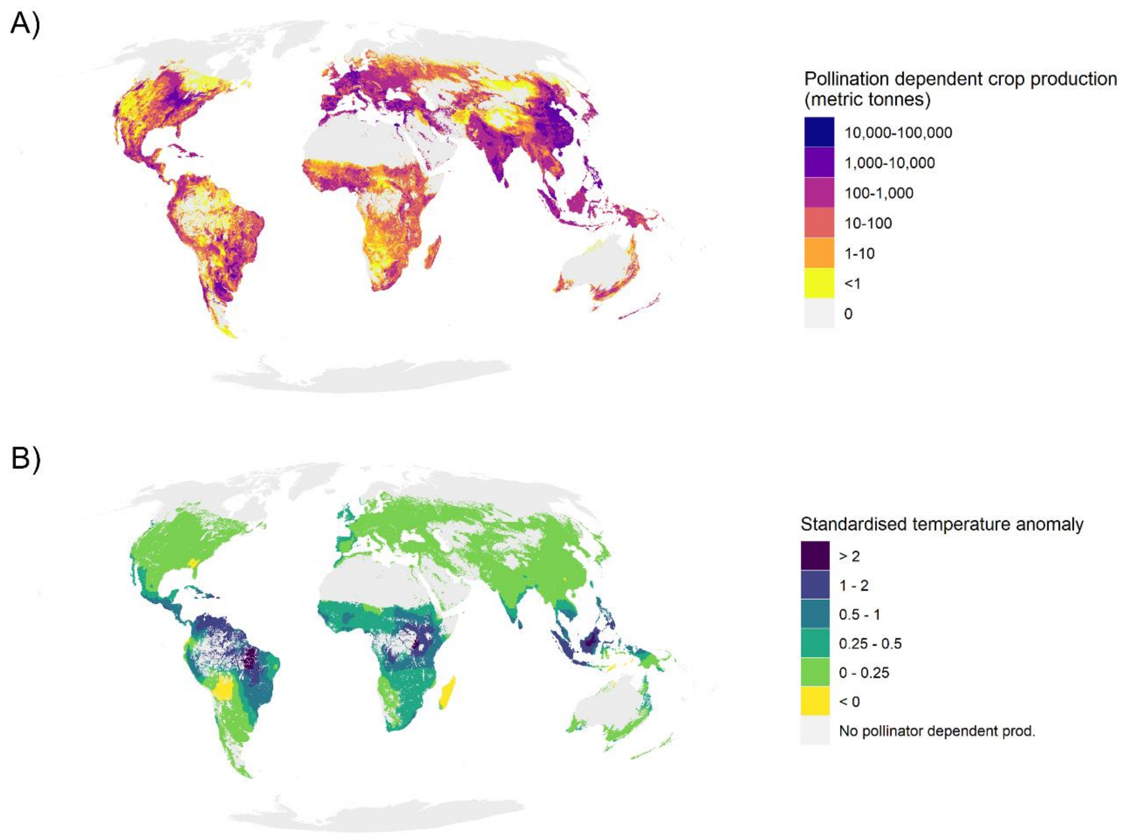

Figure S8. Spatial estimates of crop production that depends on animal pollination for the year 2000 (A), and the standardised temperature anomaly averaged for the years 2004 to 2006 (B). Both maps show only grid cells in which there is some crop production dependent on animal pollination. Animal-pollination-dependent production (A) is calculated as estimated total crop production (*47*), adjusted for the proportional dependence on animal pollination (*36*). The standardised temperature anomaly is the change in mean of monthly mean daily temperatures between a baseline period (1901-1930) and 2004-2006, divided by the standard deviation across monthly mean daily temperatures across the 30-year baseline period.

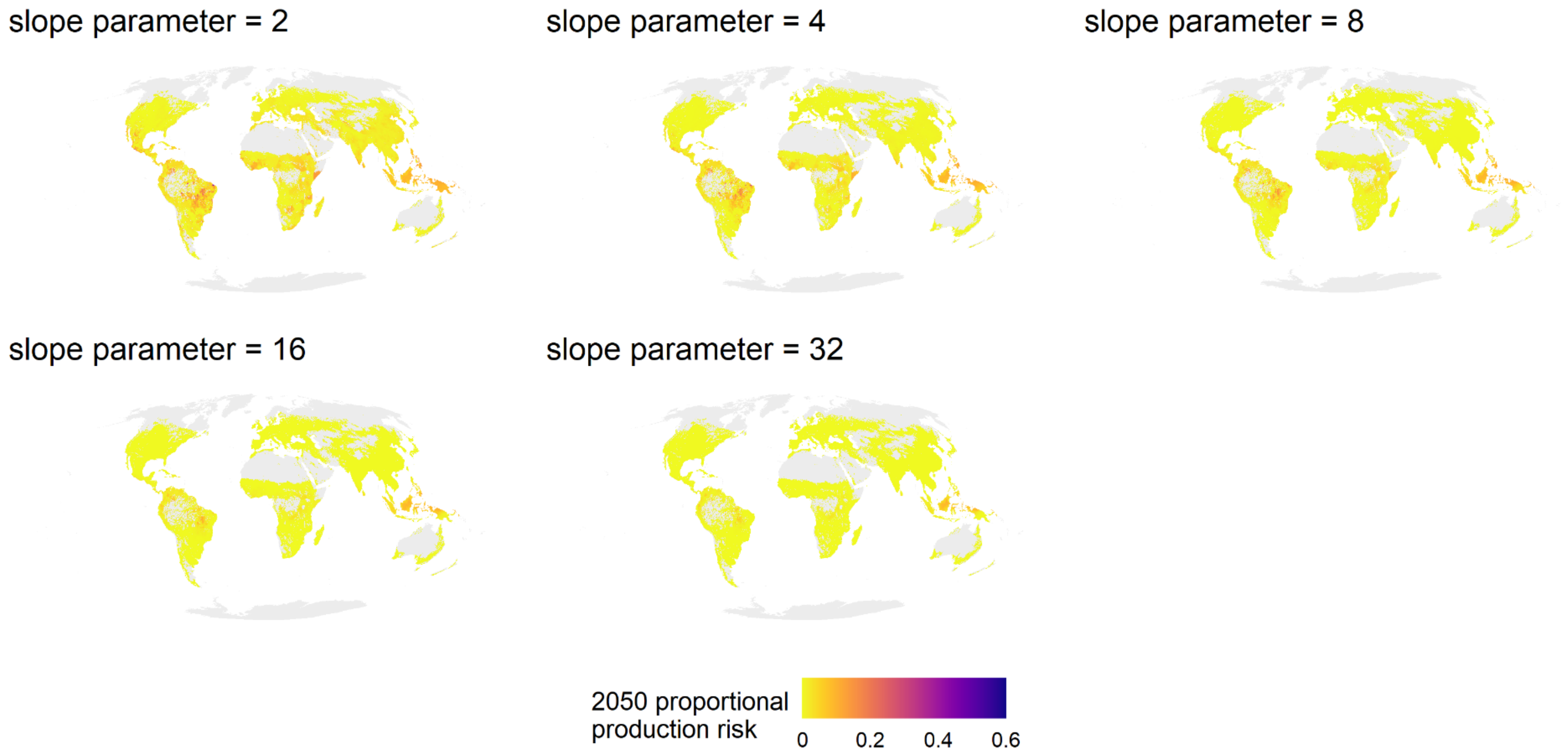

Figure S9. Sensitivity of the spatial distribution of proportional production risk to variation in the assumed relationship between pollinator abundance and production risk. Projections are shown for the RCP 6.0 climate scenario in 2050, based on temperature projections from four climate models (GFDL, HadGEM2, IPSL, and MIROC5), and for four possible relationships between pollinator abundance and production risk: linear (slope parameter = 2), and increasingly steep concave relationships (slope parameters of 4 to 32).

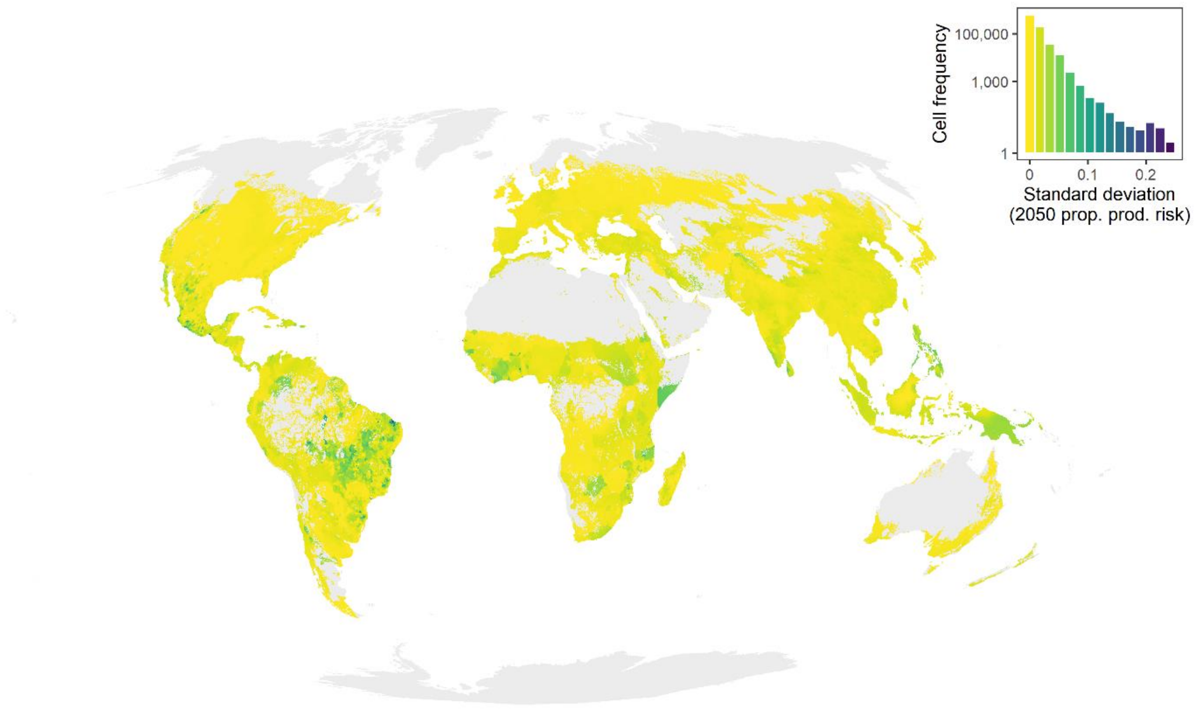

Figure S10. Variation in proportional production risk according to the assumed relationship between pollinator abundance and production risk. Projections are for the RCP 6.0 climate scenario in 2050, using temperature estimates averaged across four climate models (GFDL, HadGEM2, IPSL, and MIROC5). Variation is shown as the standard deviation among proportional risk estimates from 5 possible relationships between pollinator abundance and crop production risk: linear, and increasingly steep concave relationships (as shown in Figures S9 and S10). The inset shows the distribution of cell-level standard deviation across all cells in the map.

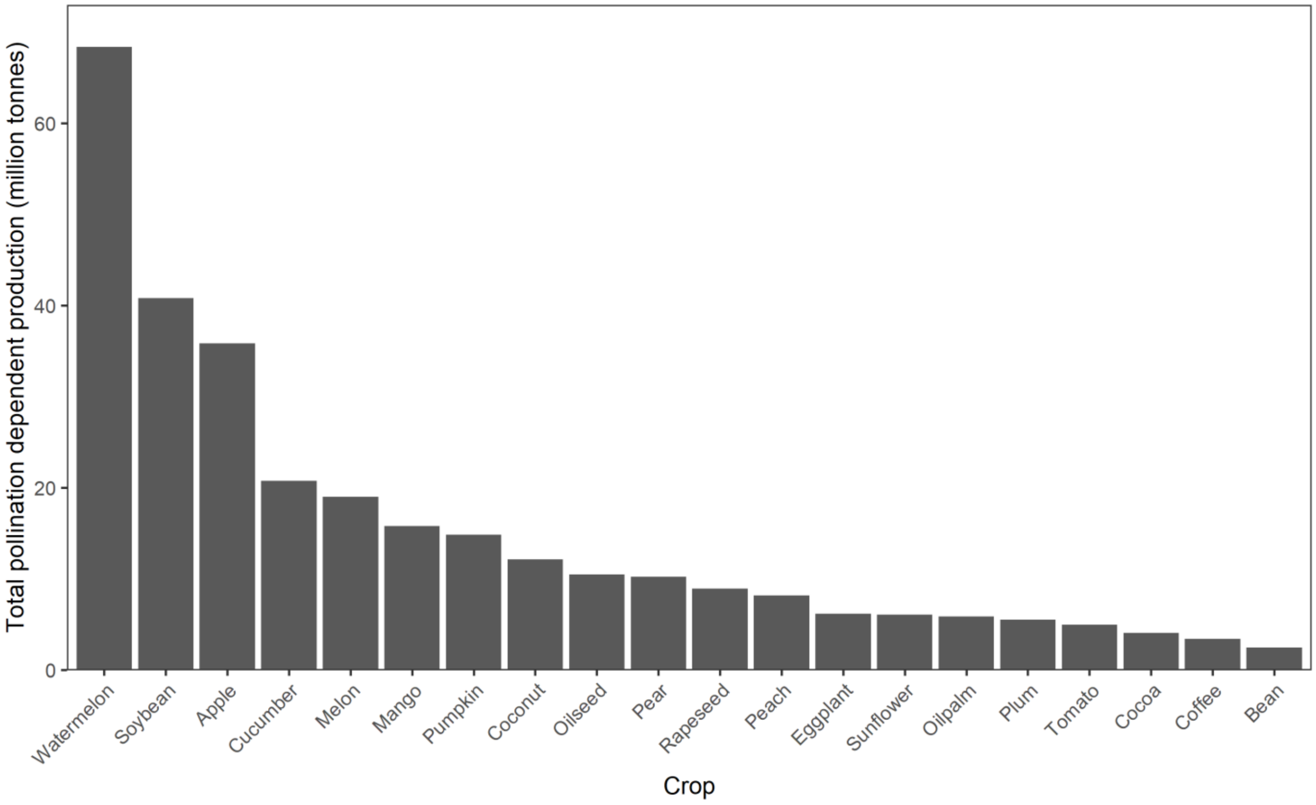

Figure S11. Total production dependent on animal pollination for the 20 crops with the highest values. Total production values are for the year 2000, taken from EarthStat (*47*). Animal-pollination-dependent production is estimated by multiplying total estimated production volume (in tonnes) for each crop by the proportional dependence on animal pollination (*36*). For any crop group in EarthStat represented by multiple estimated pollination dependencies, we took the dependence to be the mean across the individual crops.

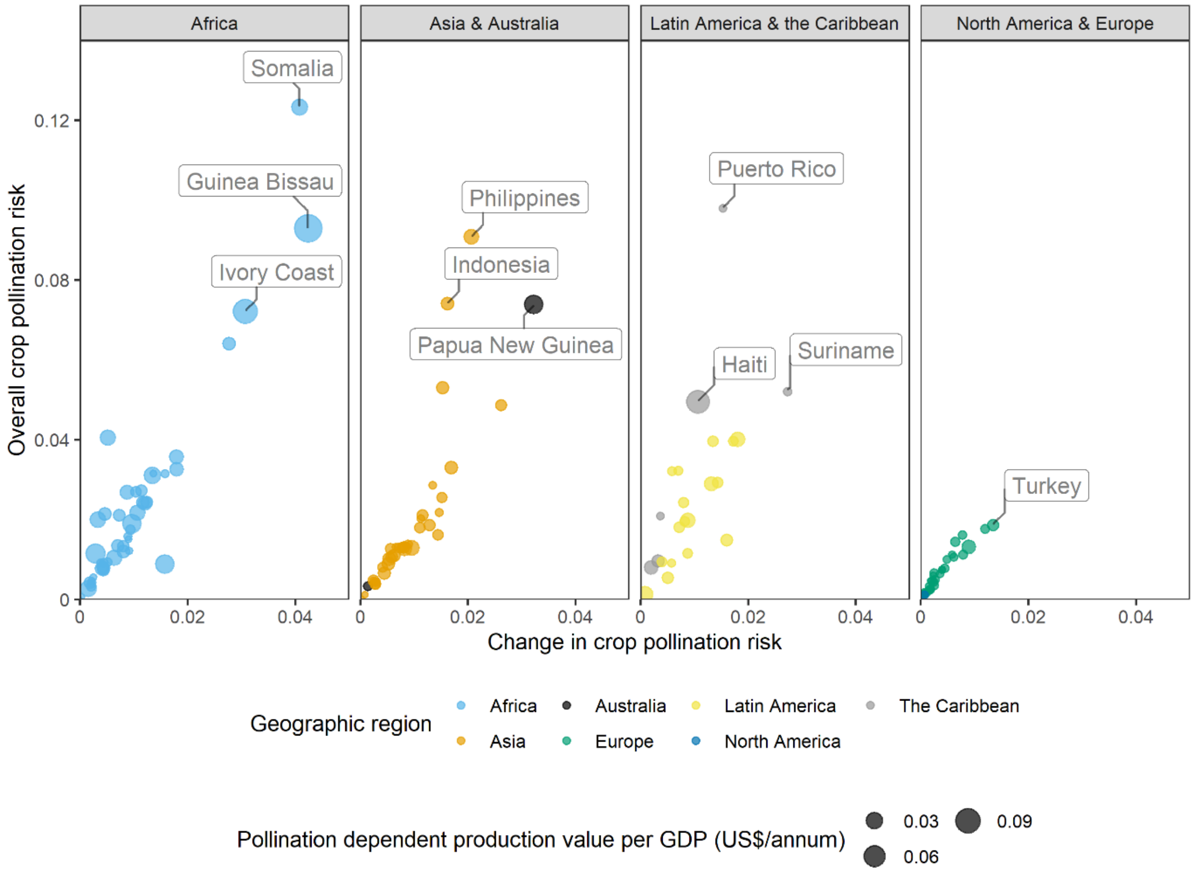

Figure S12. Proportional production risk at the level of each country in 2050 under the RCP 6.0 climate scenario, assuming a linear relationship between insect pollinator abundance loss and production loss for crops dependent on animal pollination. Here overall risk is the median of proportional production risk for all cells of that country, whilst change in risk is the difference in overall risk between the start and the end of the series. Point size here represents the total value of the pollination dependent production in that country as a proportion of GDP, calculated from the product of total pollination dependent production per annum according to (*47*) and (*36*) and the per tonne value of each crop (*91*). Colour represents the geographic region of each country, distinguishing between regions within a panel: Light blue, Africa; orange, Asia; black, Australia; green, Europe; dark blue, North America; yellow, Latin America; grey, the Caribbean.

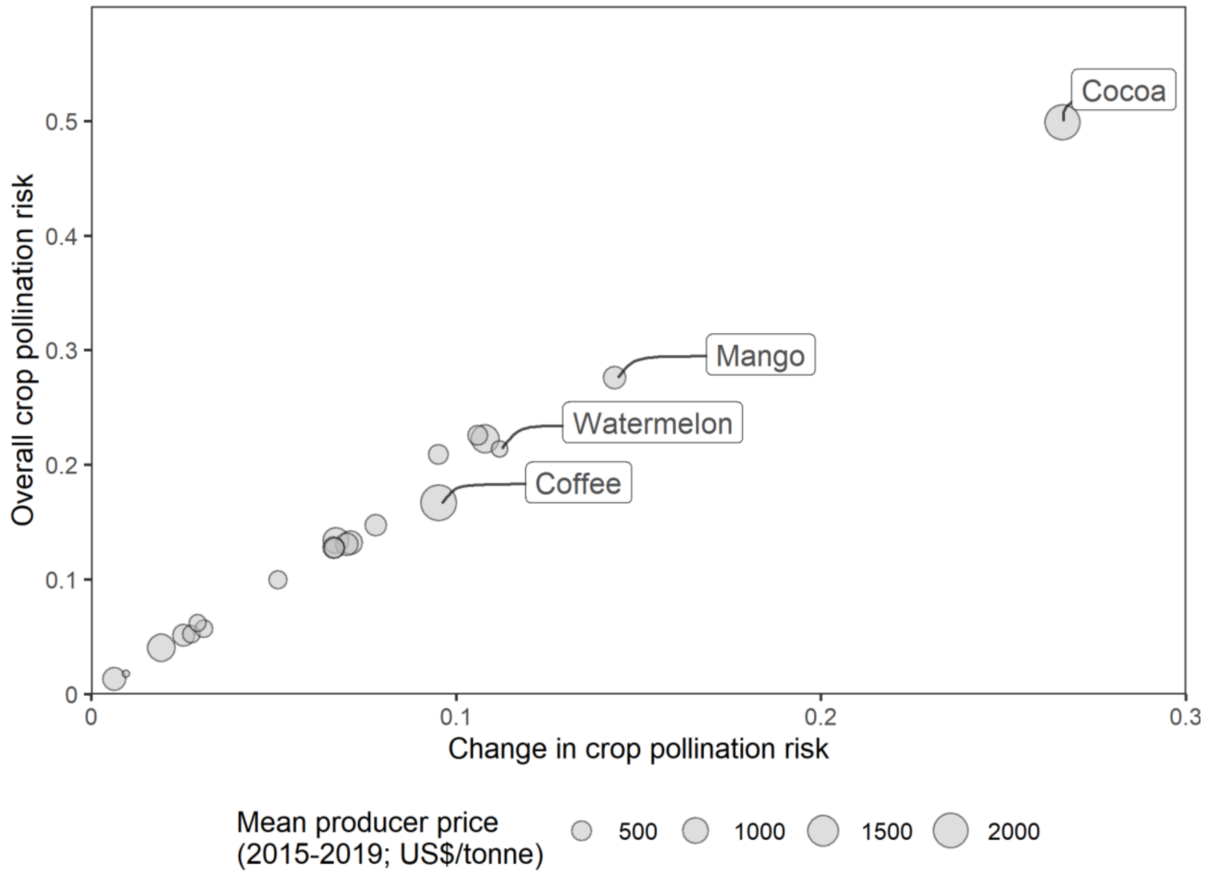

Figure S13. Proportional production risk for the 20 crops with the highest total pollination dependent production globally (see Figure S11 for the top 20 crops) in 2050 under the RCP 6.0 climate scenario, assuming a linear relationship between insect pollinator abundance loss and production loss for crops dependent on animal pollination. Overall risk here is the median of proportional production risk for all spatial cells in which that crop appears, whilst change in risk is the difference in overall risk between the start and the end of the series. Point size represents an estimation of the per tonne value of each crop for the years 2015-2019, calculated from (*91*).

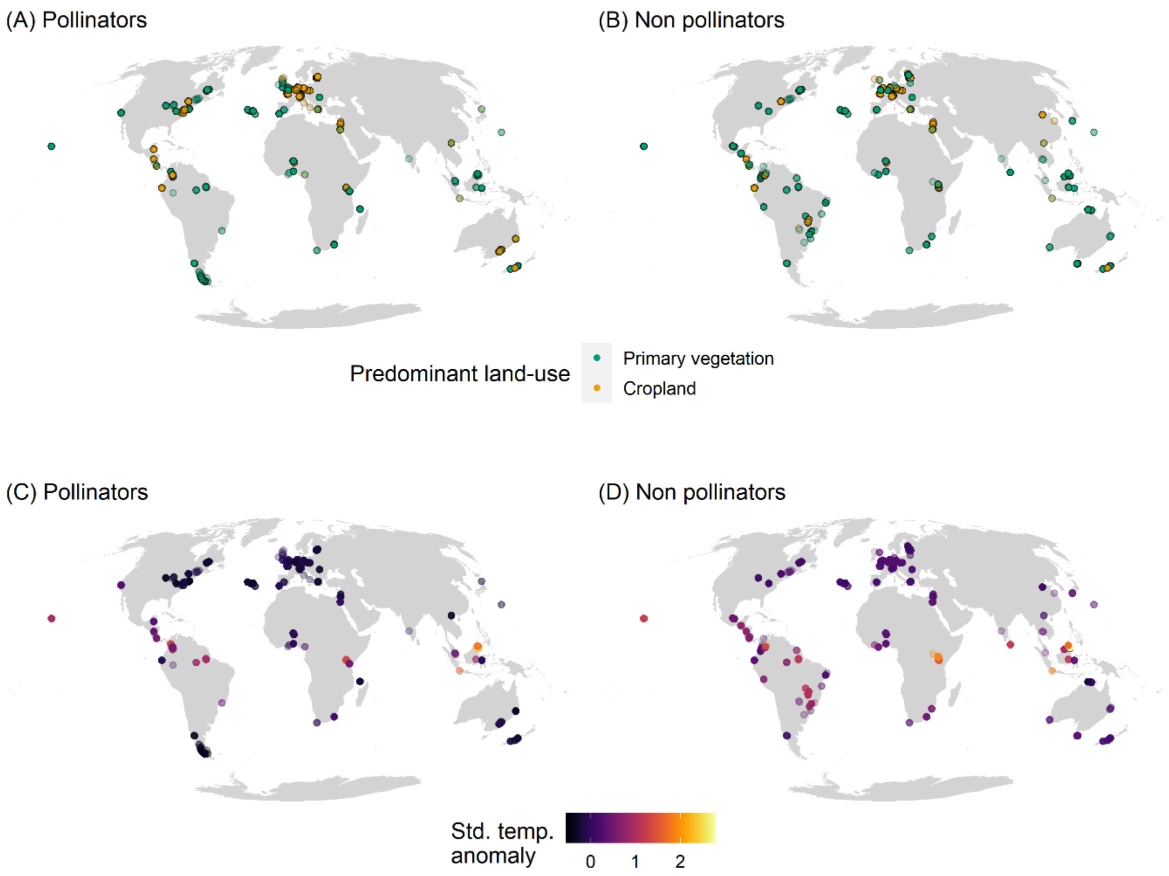

Figure S14. Spatial distribution of the sites in the PREDICTS database for both pollinating (A and C) and non-pollinating insects (B and D). In panels (A) and (B), colours indicate the distribution of sites among primary vegetation and cropland, whereas in panels (C) and (D), colours represent the standardised temperature anomaly for each site for the year of sampling.

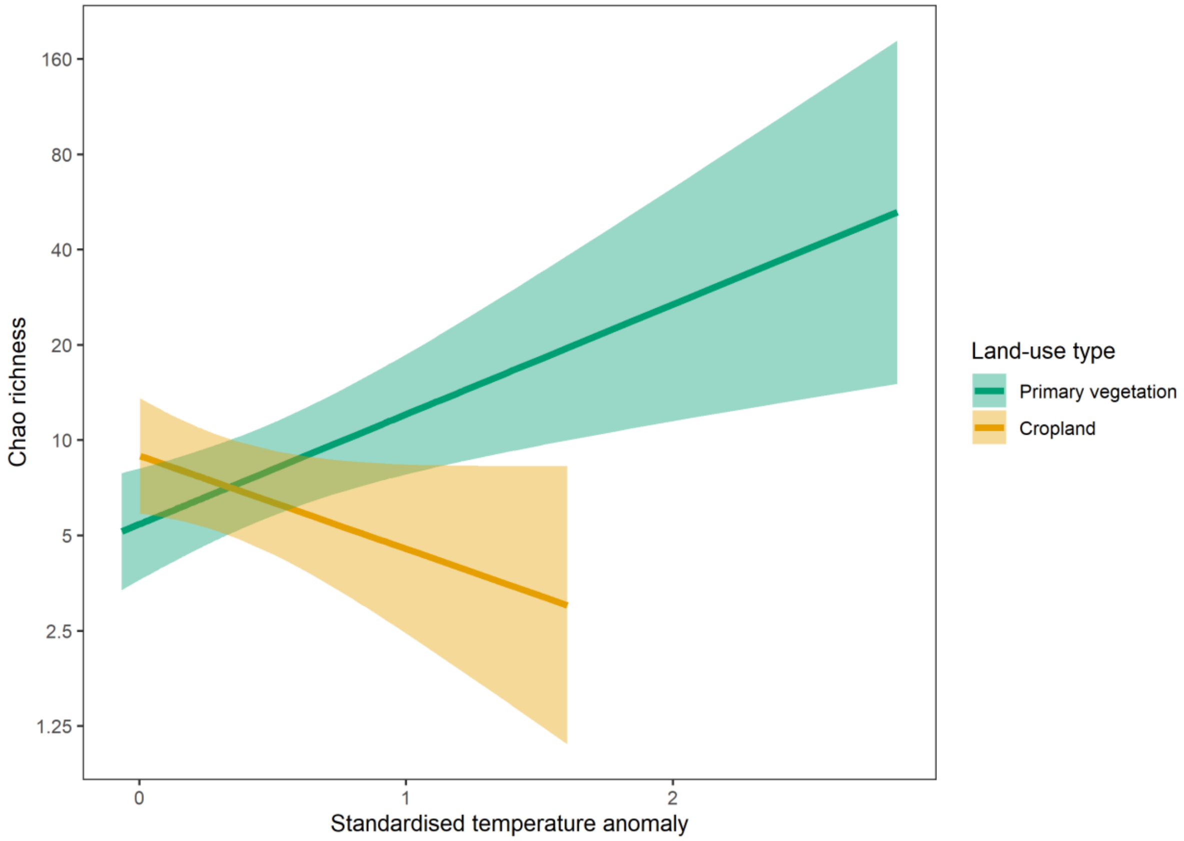

Figure S15. Response of species richness of pollinating insects (estimated using the Chao estimator) to the interactive effect of standardised temperature anomaly and land use (primary vegetation versus cropland). Note that richness is plotted on a $\log_e$ scale (although the labels are back-transformed). Results are based on generalised linear mixed-effects models with a Poisson distribution of errors. Shading represents 95% confidence intervals around the mean fitted effect. Green = primary vegetation; yellow = cropland.

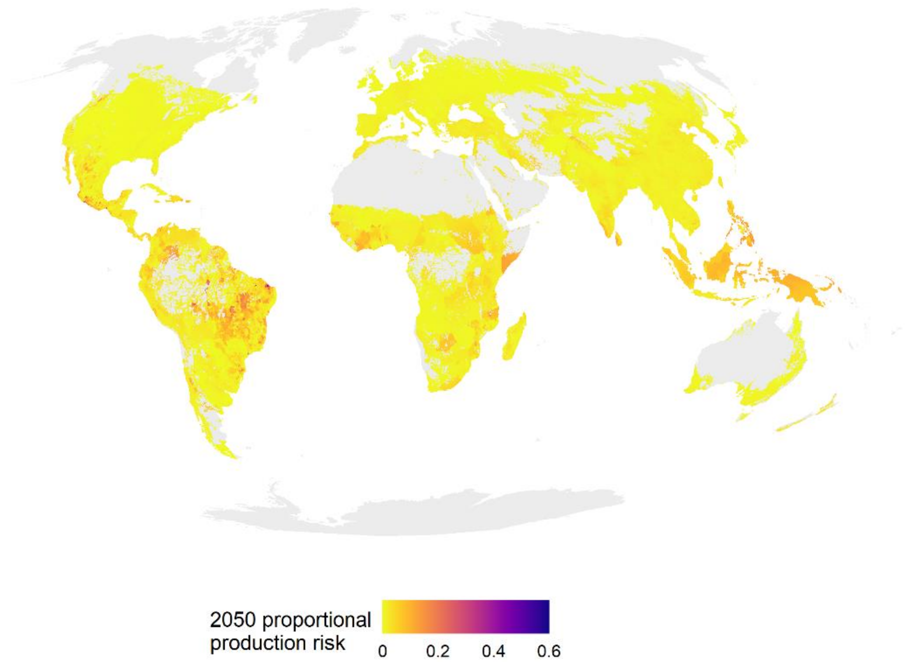

Figure S16. Projected change globally in crop production estimated to be at risk in 2050 under the RCP 6.0 climate scenario, assuming a linear relationship between species richness of insect pollinators and production loss for crops dependent on animal pollination. All projections are based on mean projections of the standardized temperature anomaly based on temperature estimates from an ensemble of individual climate models. Shown here is the sum of crop production at risk across all crops with some dependence on animal pollination, as a proportion of the production of all crops grown in a location ('proportional production risk').

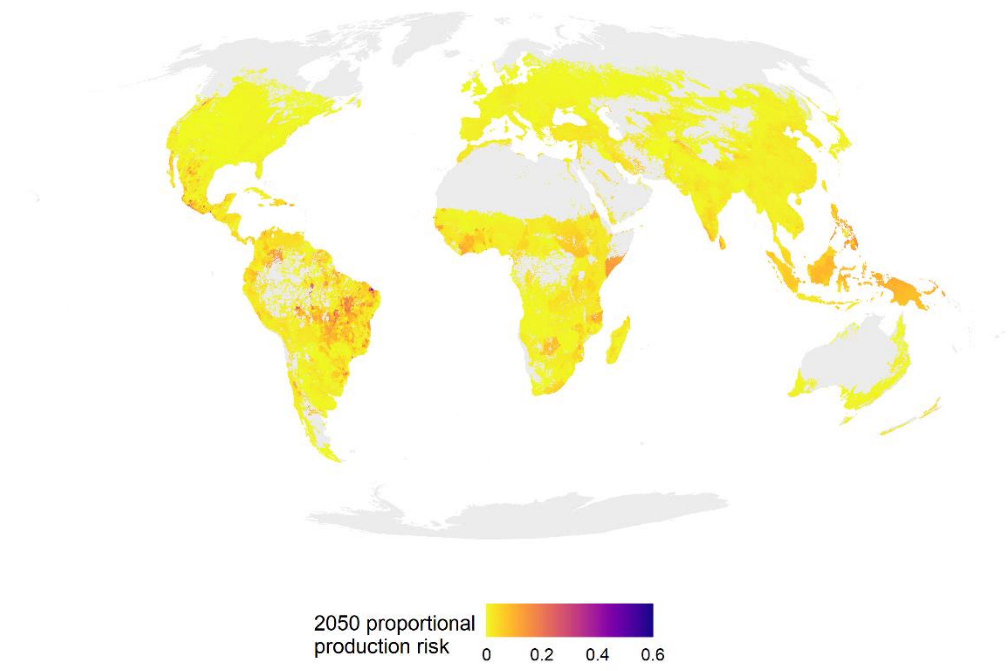

Figure S17. Projected change globally in crop production estimated to be at risk in 2050 under the RCP 6.0 climate scenario, assuming a linear relationship between bee abundance loss and production loss for crops dependent on animal pollination. All projections are based on mean projections of the standardized temperature anomaly based on temperature estimates from an ensemble of individual climate models. Shown here is sum of crop production at risk across all crops with some dependence on animal pollination, as a proportion of the production of all crops grown in a location (‘proportional production risk’).

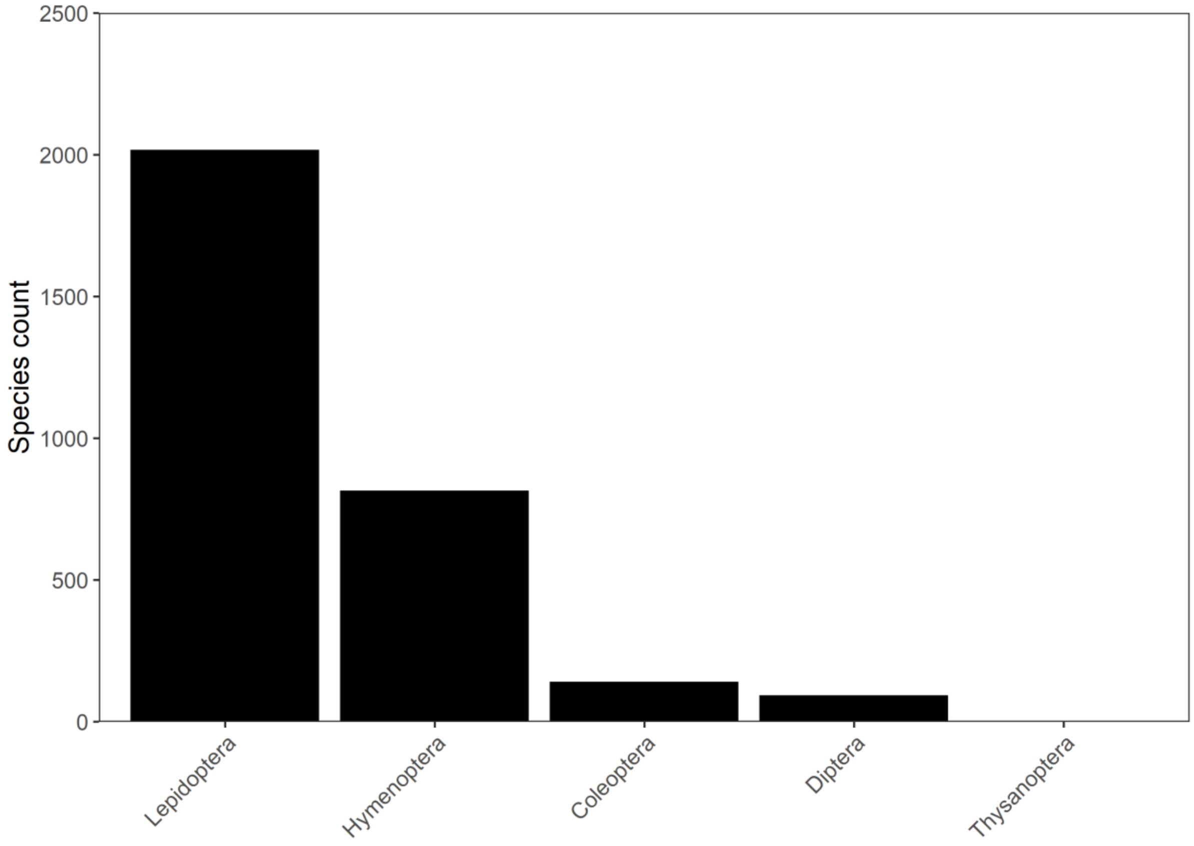

Figure S18. Frequency of insect pollinator species in the PREDICTS database for each taxonomic order.

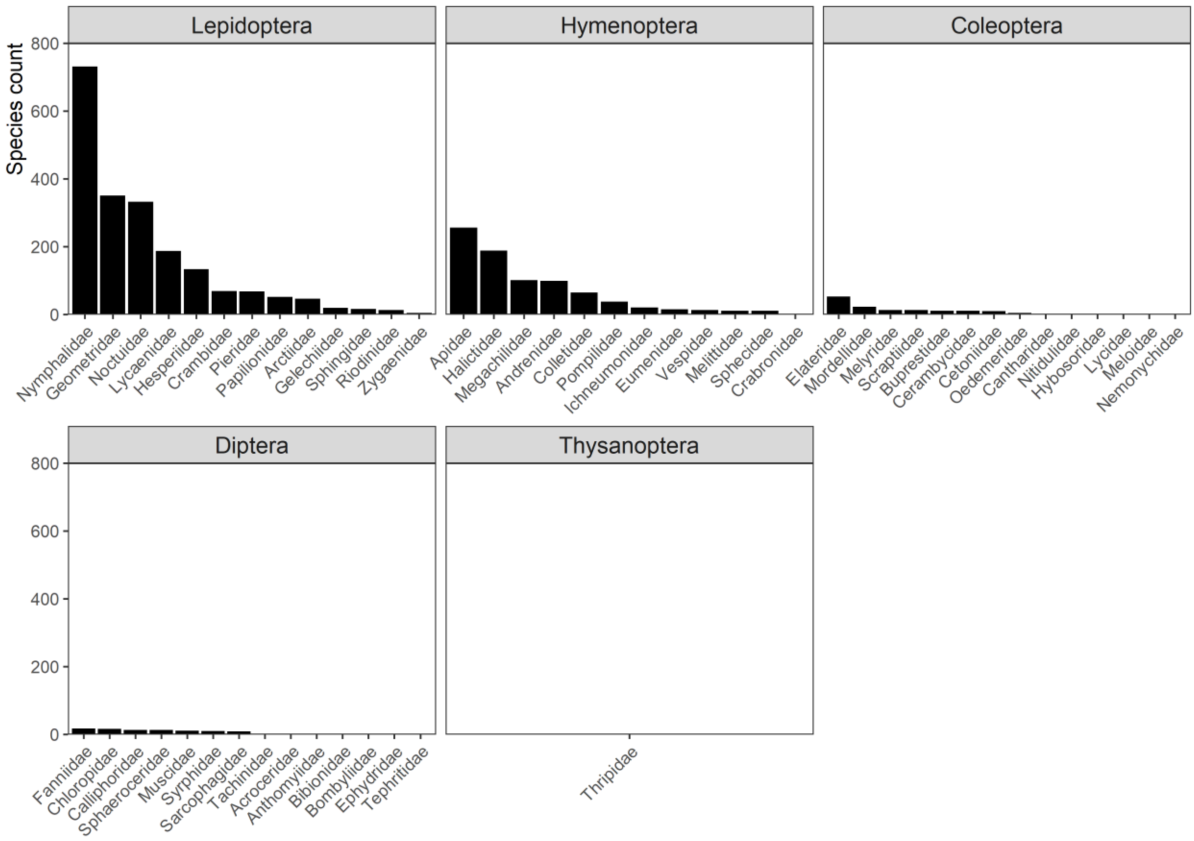

Figure S19. Frequency of insect pollinator species in the PREDICTS database for each taxonomic family.

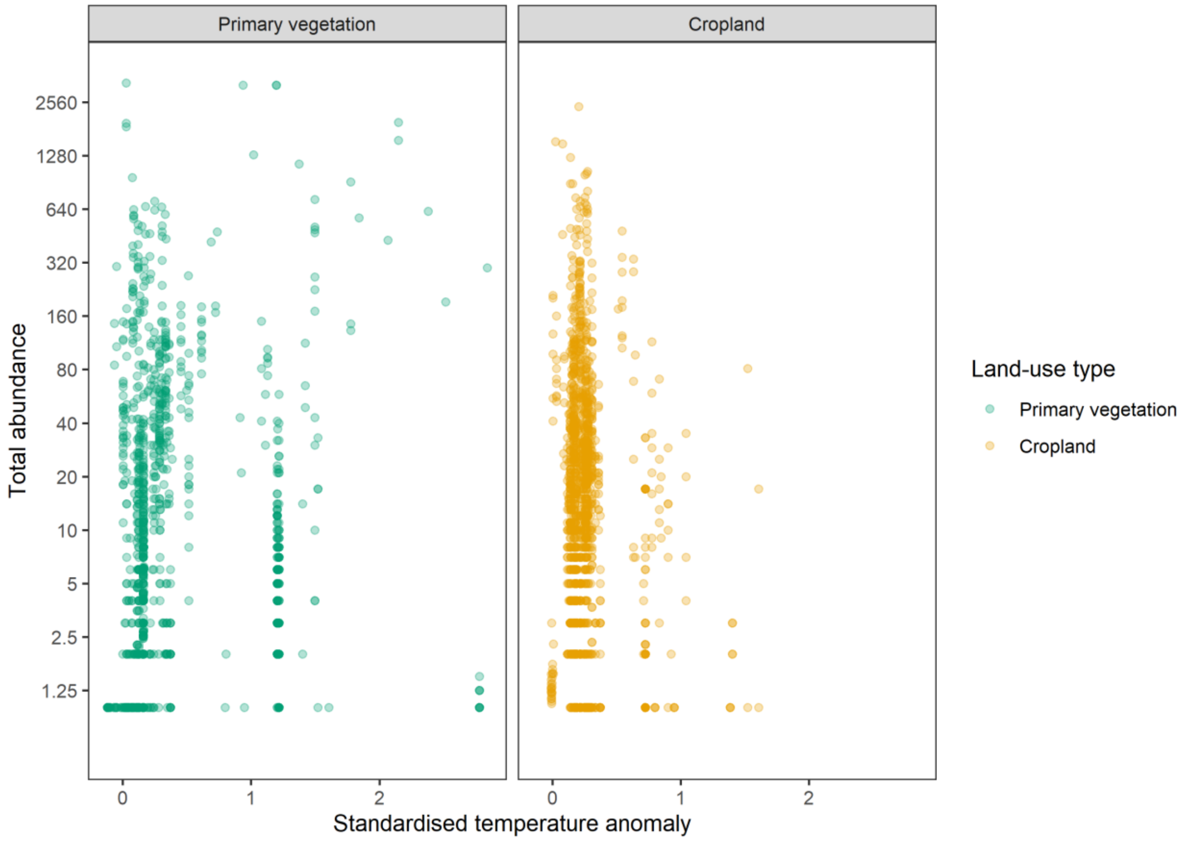

Figure S20. Sampled total abundance (+1) of insect pollinators in the PREDICTS database for primary vegetation (green, left panel) and cropland (yellow, right panel) sites, as a function of the standardised temperature anomaly at the date of sampling. Note that total abundance here is plotted on a $\log_e$ scale (although the labels are back-transformed), and that sampling method heterogeneity between studies means relationships can be difficult to infer from plotting alone.

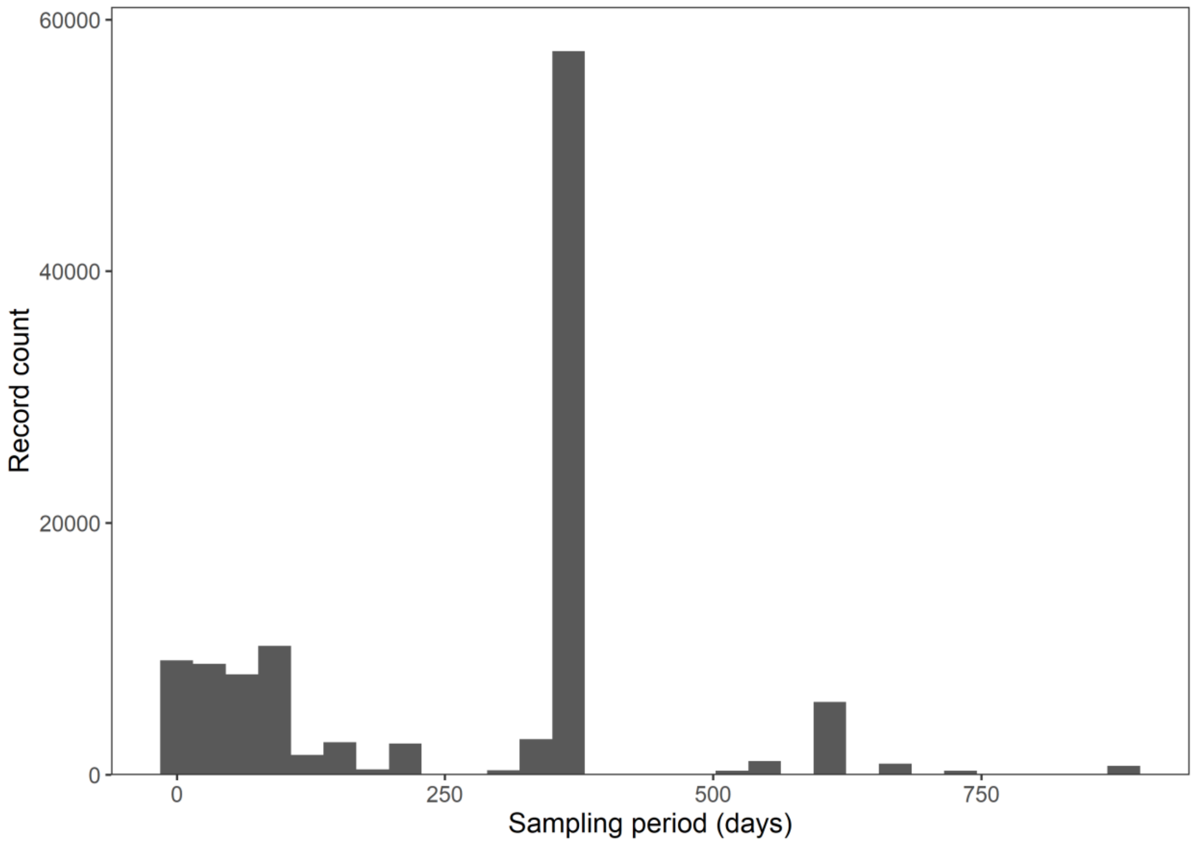

Figure S21. The distribution of sampling period for all insect pollinator records in PREDICTS. Note that only ~8% of records are for a sampling period of greater than 1 year.

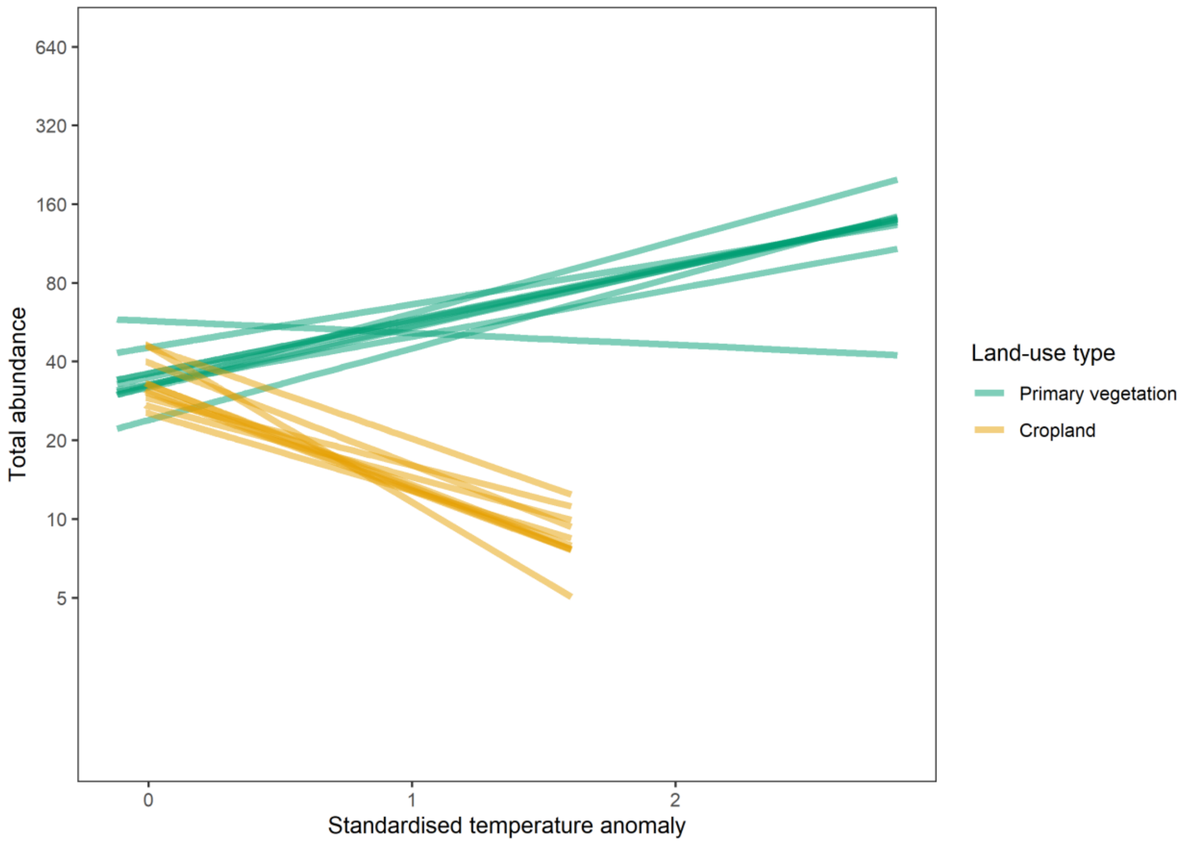

Figure S22. Response of pollinating insect total abundance to the interactive effect of standardised temperature anomaly and land use (primary vegetation versus cropland), with one of each of the top 10 sampling methods removed for each line. Note that total abundance is plotted on a $\log_e$ scale (although the labels are back-transformed). Results are based on generalised linear mixed-effects models with a Poisson distribution of errors. Green = primary vegetation; yellow = cropland.

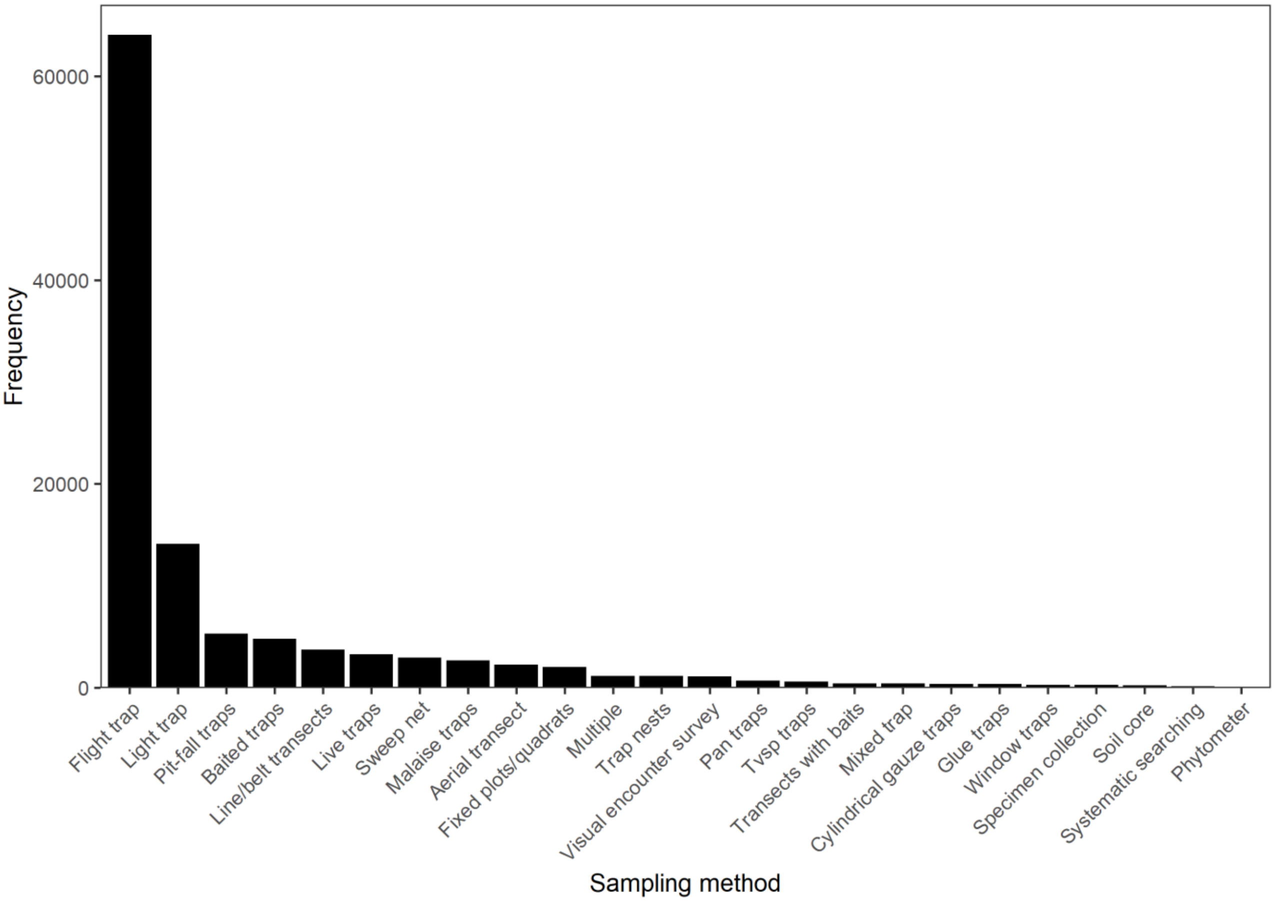

Figure S23. The frequency of records for each sampling method used to collect insect pollinator data in the PREDICTS database (total number of records equals 112555).

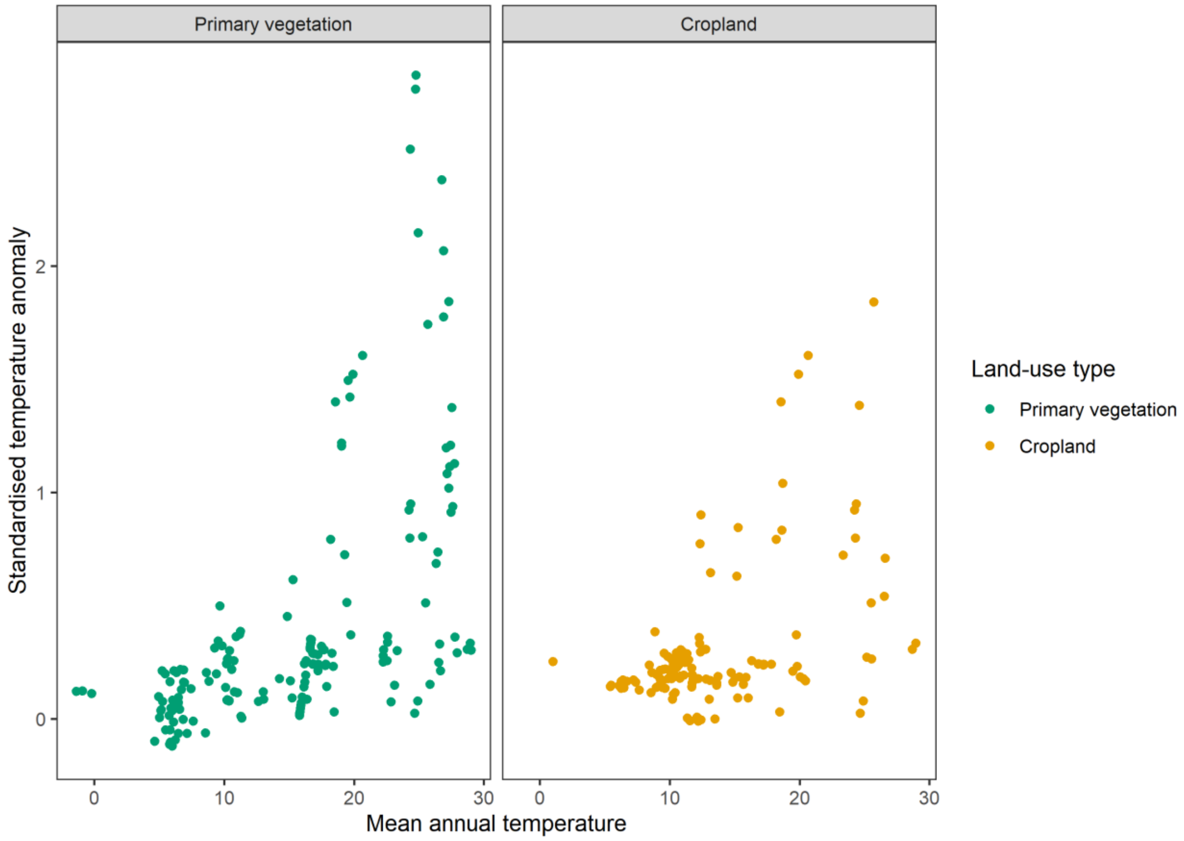

Figure S24. Mean annual temperature (°C) at the year of sampling plotted against the standardised temperature anomaly, for each site in the PREDICTS database at which some pollinating species were sampled. Left panel: primary vegetation sites (green). Right panel: cropland sites (orange).

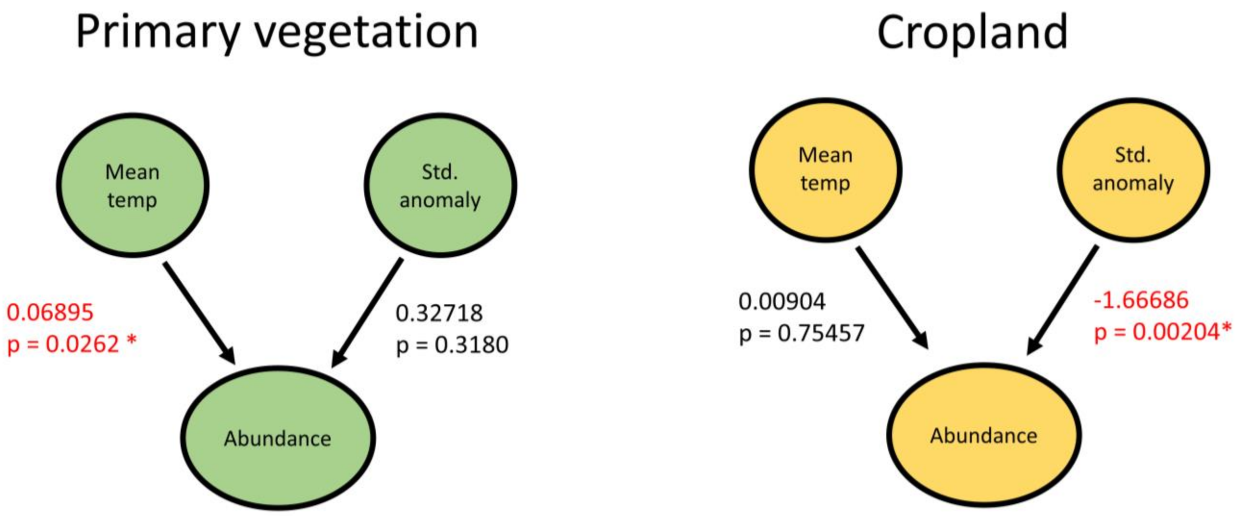

Figure S25. Simple directed acyclic graphs for the effect of mean annual temperature and standardised temperature anomaly on insect pollinator total abundance, on either primary vegetation (in green) or cropland (in yellow) sites. Numbers beside each arrow represent the coefficients (top) and p values (bottom) for that fixed effect, derived from a mixed-effects linear model predicting log(total abundance + 1) as a function of standardised temperature anomaly and mean annual temperature, with the random intercepts study (SS) and block (SSB). Significant effects are indicated in red, and nonsignificant in black.

Tables

Table S1. The total number of cropland and primary vegetation sites for pollinating and likely non-pollinating insects in the PREDICTS database.

**Land-use type**	**Pollinator status**	**N (sites)**
Primary vegetation	Pollinators	1166
Cropland	Pollinators	1507
Primary vegetation	Likely non-pollinators	1747
Cropland	Likely non-pollinators	922

Table S2. The total number of species in the PREDICTS database for pollinating and likely non-pollinating insects, sampled in either cropland or primary vegetation.

**Order**	**Pollinator status**	**N (species)**
Lepidoptera	Pollinators	2018
Hymenoptera	Pollinators	815
Coleoptera	Pollinators	152
Diptera	Pollinators	94
Thysanoptera	Pollinators	1
Lepidoptera	Likely non-pollinators	310
Hymenoptera	Likely non-pollinators	475
Coleoptera	Likely non-pollinators	1674
Diptera	Likely non-pollinators	317
Thysanoptera	Likely non-pollinators	4
Hemiptera	Likely non-pollinators	120
Odonata	Likely non-pollinators	80
Orthoptera	Likely non-pollinators	71
Isoptera	Likely non-pollinators	33
Psocodea	Likely non-pollinators	12
Archaeognatha	Likely non-pollinators	2
Blattodea	Likely non-pollinators	2
Dermaptera	Likely non-pollinators	2
Neuroptera	Likely non-pollinators	1
Trichoptera	Likely non-pollinators	1

Table S3. AIC and R^2 values for linear mixed-effects models with different random-effects structures fitting $\log_e$ total abundance as a function of land-use type (cropland and primary vegetation), standardised climate anomaly, and their interaction, for both pollinating and likely non-pollinating insect species. Random-effects structures considered were either study identity, or study identity and spatial block nested within study.

**Model**	**Pollinator status**	**AIC**	**R^2 (marginal)**	**R^2 (conditional)**
log(Total_abundance) ~ standard_anom * land_use +	Pollinators	7171.79	0.017	0.823
log(Total_abundance) ~ standard_anom * land_use + (1|SS) + (1|SSB)	Pollinators	7042.814	0.023	0.849
log(Total_abundance) ~ standard_anom * land_use +	Likely non-	6379.434	0.017	0.902
log(Total_abundance) ~ standard_anom * land_use + (1|SS) + (1|SSB)	Likely non-pollinators	6300.413	0.018	0.912

Table S4. Proportional dependence on animal pollination for the crops/crop groups mapped in EarthStat (*47*). For each crop group, we show the average and standard error of proportional pollination dependence estimates (*36*) among individual crops, a crop common name, and whether artificial-pollination is known to occur in that crop, according to (*61*). Standard errors are shown as 'NA' where there was a one-to-one match between crops in EarthStat and crops with estimated dependence on animal pollination. 'Monfreda crop group' is the exact string used in (*47*) for each crop raster layer.

Monfreda crop group	Crop common name	Mean pollination dependence	Standard error	Artificial pollination documented in multiple localities
brazil	Brazil nut	0.95	NA	
cocoa	Cocoa bean	0.95	NA	
kiwi	Kiwi fruit	0.95	NA	Y
melonetc	Other melons (inc. cantaloupes)	0.95	NA	
nutnes	Nuts (not elsewhere)	0.95	NA	
pumpkinetc	Pumpkin, squash and gourds	0.95	NA	
vanilla	Vanilla	0.95	NA	Y
watermelon	Watermelon	0.95	NA	
almond	Almond	0.65	NA	
aniseetc	Anise, badian, fennel, coriander	0.65	0	
apple	Apple	0.65	NA	Y
apricot	Apricot	0.65	NA	
avocado	Avocado	0.65	NA	
blueberry	Blueberry	0.65	NA	
buckwheat	Buckwheat	0.65	NA	
cashew	Cashew nut	0.65	NA	
cashewapple	Cashewapple	0.65	NA	
cherry	Cherry	0.65	0	
cranberry	Cranberry	0.65	NA	
cucumberetc	Cucumbers and gherkins	0.65	NA	
mango	Mango	0.65	NA	
nutmeg	Nutmeg	0.65	NA	
peachetc	Peaches and nectarines	0.65	NA	
pear	Pear	0.65	NA	

plum	Plum	0.65	NA	
rasberry	Raspberry	0.65	NA	
berrynes	Berries (not elsewhere)	0.616667	0.202759	
tropicalnes	Tropical fresh fruits (not elsewhere)	0.531818	0.111043	
fruitnes	Fresh fruits (not elsewhere)	0.51	0.16	
coffee	Coffee	0.45	NA	
spicenes	Spices (not elsewhere)	0.45	0.2	
oilseednes	Oilseed (not elsewhere)	0.316667	0.176383	
broadbean	Broadbean	0.25	0	
chestnut	Chestnut	0.25	NA	
coconut	Coconut	0.25	NA	
currant	Currant	0.25	NA	
eggplant	Eggplant	0.25	NA	
fig	Fig	0.25	NA	
mustard	Mustard seed	0.25	NA	
oilseedfor	Oilseed (forage)	0.25	NA	
okra	Okra	0.25	NA	
rapeseed	Rapeseed	0.25	NA	
sesame	Sesame seed	0.25	NA	
soybean	Soybean	0.25	NA	
stonefruitnes	Stonefruits (not elsewhere)	0.25	NA	
strawberry	Strawberry	0.25	NA	
sunflower	Sunflower seed	0.25	NA	
bean	Beans (dry)	0.15	0.1	
chilleetc	Chillies and peppers	0.05	NA	
citrusnes	Citrus fruits (not elsewhere)	0.05	NA	
greenbroadbean	Leguminous vegetables (not elsewhere)	0.05	NA	
groundnut	Groundnuts (with shells)	0.05	NA	
lemonlime	Lemons and limes	0.05	NA	
oilpalm	Oilpalm	0.05	NA	Y
papaya	Papaya	0.05	NA	
pea	Peas (dry)	0.05	0	
persimmon	Persimmon	0.05	NA	
pigeonpea	Pigeon pea	0.05	NA	
safflower	Safflower	0.05	NA	
tomato	Tomato	0.05	NA	Y

chickpea	Chickpea	0	NA	
grape	Grape	0	NA	
greenpea	Greenpea	0	NA	
lentil	Lentil	0	NA	
olive	Olive	0	NA	
pepper	Pepper	0	NA	
quinoa	Quinoa	0	NA	

Table S5. Model summary for a mixed-effects linear model predicting log(total abundance + 1) as a function of standardised temperature anomaly, land-use (cropland or primary vegetation), and an interaction between land-use and standardised temperature anomaly, with the random intercepts study (SS) and block (SSB).

Fixed effects	Estimate	Standard error	p value
Intercept	3.53640	0.23433	$< 2 \times 10^{-16}$
Standardised temperature anomaly	0.49520	0.28177	0.0795
Predominant land use (Cropland)	-0.06065	0.14287	0.6712
Standardised temperature anomaly : Predominant land use (Cropland)	-1.37805	0.29112	2.33×10^{-6}

Table S6. Model summary for a mixed-effects linear model predicting log(total abundance + 1) as a function of standardised temperature anomaly, mean annual temperature, land-use (cropland or primary vegetation), an interaction between mean annual temperature and land-use, and an interaction between standardised temperature anomaly and land-use, with the random intercepts study (SS) and block (SSB).

**Fixed effects**	**Estimate**	**Standard error**	**p value**
Intercept	1.37443	0.42356	0.00138
Mean annual temperature	0.11762	0.02343	8.8×10^{-7}
Standardised temperature anomaly	0.15796	0.29124	0.58777
Predominant land use (Cropland)	2.85321	0.35376	1.37×10^{-15}
Mean annual temperature : Predominant land use (Cropland)	-0.14633	0.01635	$< 2 \times 10^{-16}$
Standardised temperature anomaly : Predominant land use (Cropland)	-0.86479	0.29341	0.00323
